# Single-cell transcriptomics reveals gene expression dynamics of human fetal kidney development

**DOI:** 10.1371/journal.pbio.3000152

**Published:** 2019-02-21

**Authors:** Mazène Hochane, Patrick R. van den Berg, Xueying Fan, Noémie Bérenger-Currias, Esmée Adegeest, Monika Bialecka, Maaike Nieveen, Maarten Menschaart, Susana M. Chuva de Sousa Lopes, Stefan Semrau

**Affiliations:** 1 Leiden Institute of Physics, Leiden University, Leiden, The Netherlands; 2 Department of Anatomy and Embryology, Leiden University Medical Center, Leiden, The Netherlands; 3 Department of Reproductive Medicine, Ghent University Hospital, Ghent, Belgium; University of Cambridge, UNITED KINGDOM

## Abstract

The current understanding of mammalian kidney development is largely based on mouse models. Recent landmark studies revealed pervasive differences in renal embryogenesis between mouse and human. The scarcity of detailed gene expression data in humans therefore hampers a thorough understanding of human kidney development and the possible developmental origin of kidney diseases. In this paper, we present a single-cell transcriptomics study of the human fetal kidney. We identified 22 cell types and a host of marker genes. Comparison of samples from different developmental ages revealed continuous gene expression changes in podocytes. To demonstrate the usefulness of our data set, we explored the heterogeneity of the nephrogenic niche, localized podocyte precursors, and confirmed disease-associated marker genes. With close to 18,000 renal cells from five different developmental ages, this study provides a rich resource for the elucidation of human kidney development, easily accessible through an interactive web application.

## Abstract 2

A detailed transcriptomic characterization of the human fetal kidney at the single-cell level allows the identification of 22 cell types and a host of novel marker genes, revealing heterogeneity in the nephrogenic niche and localized podocyte precursors.

## Author summary

Regenerative medicine offers an exciting avenue to potential cures of kidney disease. However, a detailed knowledge of the structure and embryonic development of the kidney is crucial, both for stimulating regeneration in the body and for growing healthy kidney tissue in a dish. Most of such knowledge has been obtained from mice, whose development differs in crucial ways from that of humans. We therefore studied the composition of human fetal kidney tissue from five developmental ages, using a technique that can measure gene expression in individual cells. Our measurements revealed 22 distinguishable cell types, some of which we localized in the tissue by fluorescence microscopy. We found several subpopulations of nephron progenitors—cells that give rise to the nephron, the functional unit of the kidney. Our study also focused on the development of podocytes, a cell type that is crucial for the filtration function of the kidney, and our results might inform attempts to recreate these cells in a dish. We hope that our data set, made conveniently accessible through a web application, will help scientists develop new regenerative medicine approaches to kidney disease.

## Introduction

**Fig 1:**
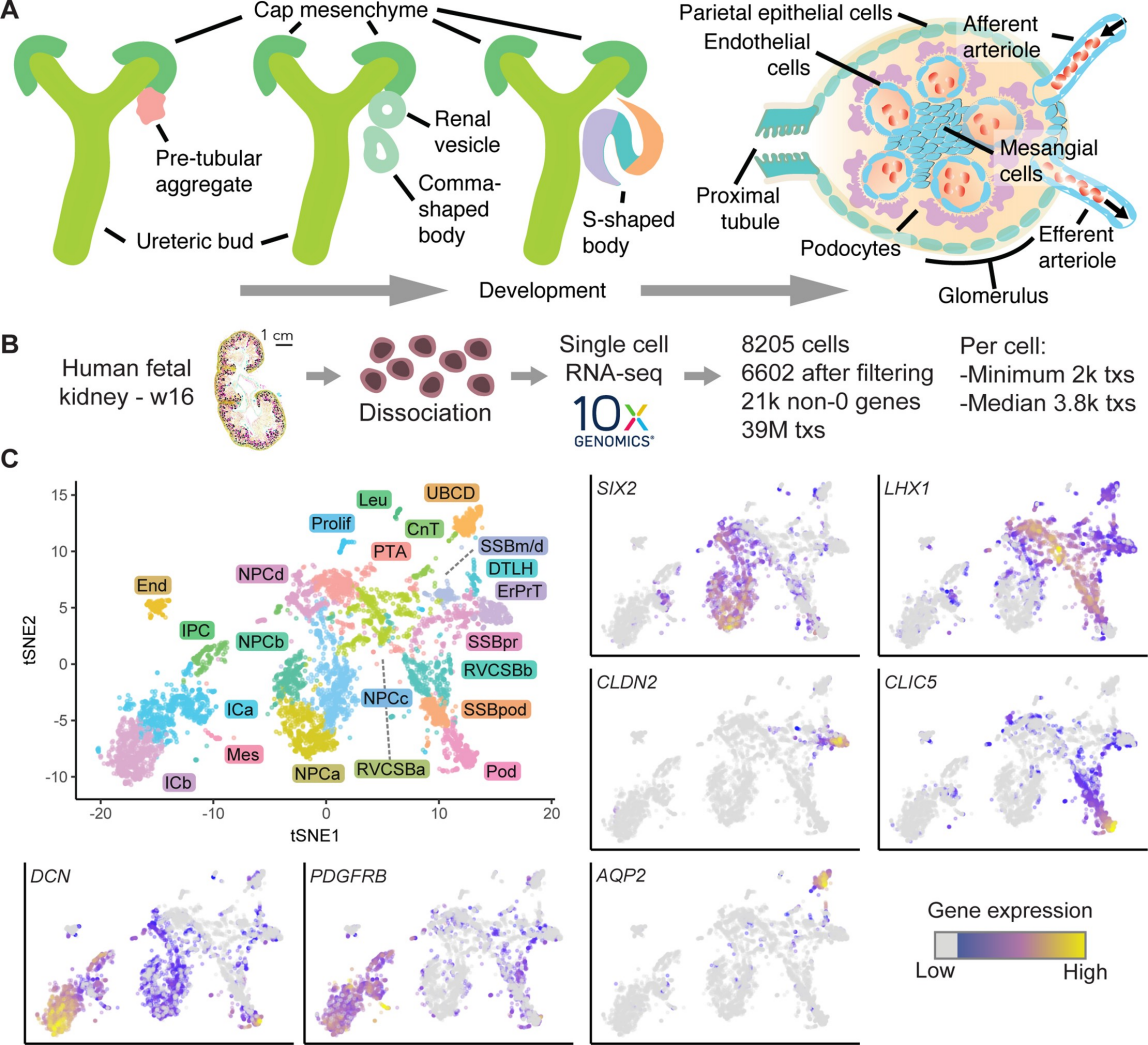
Single-cell transcriptomics identified 22 unique cell types in the human fetal kidney. (A) Schematic of kidney epithelium development. (B) Overview of the scRNAseq experiment. (C) Top left: 2D tSNE map of 6,602 human fetal kidney cells. Colors and labels indicate the assigned cell type. (Other panels) tSNE maps indicating expression of *SIX2*, *LHX1*, *CLDN2*, *CLIC5*, *DCN*, *PDGFRB*, and *AQP2*. Expression is indicated by color; expression values of 1 are plotted in gray. The numerical data underlying this figure can be found in S1 Data. CnT, connecting tubule; DTLH, distal tubule/loop of Henle; End, endothelial cells; ErPrT, early proximal tubule; ICa, interstitial cells a; ICb, interstitial cells b; IPC, interstitial progenitor cell; Leu, leukocyte; Mes, mesangial cell; NPCa, nephron progenitor cells a; NPCb, nephron progenitor cells b; NPCc, nephron progenitor cells c; NPCd, nephron progenitor cells d; Pod, podocyte; Prolif, proliferating cells; PTA, pretubular aggregate; RVCSBa, renal vesicle/comma-shaped body a; RVCSBb, renal vesicle/comma-shaped body b; scRNA seq, single-cell RNA sequencing; SSBm/d, s-shaped body medial/distal; SSBpod, s-shaped body podocyte precursor cells; SSBpr, s-shaped body proximal precursor cells; tSNE, t-distributed stochastic neighbor embedding; tx, transcript; UBCD, ureteric bud/collecting duct; w16, week 16.

Mammalian kidney development initiates in the intermediate mesoderm through crosstalk between the metanephric mesenchyme (MM) and the ureteric bud (UB). The UB originates from the nephric duct, invades the MM, and starts to subdivide progressively into multiple ramifications. The UB tip cells, which make the first contact with the MM, become enveloped by an assembly of mesenchymal cells, the cap mesenchyme (CM) (Fig 1A and S1 Fig). The CM contains nephron progenitor cells (NPCs), which give rise to the whole nephron epithelium through tightly regulated morphogenic transformations [1]. Self-renewal of (mouse) NPCs is governed by key transcription factors, such as *Six2* and *Meox1*, which mark the nephrogenic zone of the kidney [2]. Signaling between UB tip cells and NPCs regulates the balance between self-renewal and differentiation of NPCs [3]. In humans, about 1 million nephrons are produced before the NPC population is irrevocably exhausted a few weeks before birth [4]. During nephrogenesis, NPCs undergo mesenchymal-epithelial transition and differentiate into a succession of intermediate structures: the pretubular aggregate (PTA), renal vesicle (RV), and comma- and s-shaped body (CSB and SSB, respectively). Then, via the capillary loop stage, mature and functional glomerular and tubular structures are eventually formed. In contrast to the nephron epithelium, the collecting duct system originates from the UB. Glial cell-derived neurotrophic factor/ RET proto-oncogene (GDNF/RET) signaling between the UB and CM critically regulates proliferation of UB tip cells and branching morphogenesis of the UB [5]. Stromal cells—such as interstitial cells (ICs), mesangial cells, juxtaglomerular cells, smooth muscle cells, fibroblasts, and pericytes—derive from a common interstitial progenitor [6,7]. Finally, vascular endothelial cells and the highly specified glomerular endothelium originate from the MM [8], and leukocytes and erythrocytes enter with the blood stream. The current understanding of mammalian kidney development is largely based on mouse studies, although it is clear that human and mouse kidneys are morphologically different. Three recent landmark studies have revealed, in great detail, a significant divergence between mouse and human renal embryogenesis in terms of morphology as well as gene expression [9–11]. These studies underline that the prevailing lack of data on human kidney development severely hinders the detailed understanding of human kidney development and possible developmental origins of kidney disease.

In the study described here, we used single-cell RNA sequencing (scRNA-seq) to study gene expression dynamics in human fetal kidney development. Analysis of a fetal kidney from week 16 (w16) of gestation revealed 22 cell types, which we identified by known marker genes. Pseudotime analysis clarified their temporal relationship. We further defined specifically expressed cell type marker genes of which many have not been implied in kidney development. Comparison to four additional samples (from w9, w11, w13, and w18) suggested that most cell types have a constant expression pattern, with the notable exception of podocytes. To highlight two ways in which our data set can be interrogated, we then explored the nephrogenic niche and the development of podocytes. Gene expression differences between four NPC clusters were related to spatial heterogeneity by immunostaining and single-molecule fluorescence in-situ hybridization (smFISH). Expression of the disease-associated gene *UNCX* was localized to NPCs and their early derivatives. Finally, we focused on podocyte development, which proceeds via a distinct precursor state. By immunostaining and smFISH, we localized these precursors in situ and confirmed the disease-associated gene *OLFM3* as a marker.

## Results

### Clustering and identification of cell types

We performed single-cell transcriptomics on a human fetal kidney from w16 of gestation, equivalent to 14 weeks of development (Fig 1B). After data pruning and stringent removal of cells affected by stress (S2 Fig, Methods), 6,602 cells were retained for further analysis. Clusters of cells were identified by hierarchical clustering after k-nearest neighbor smoothing [12]. We assigned cell types to these clusters by expression of marker genes from the literature on mouse kidney development. The studies that linked the genes of this literature set to particular cell types are referenced in S1 Table. After merging similar clusters (S3 Fig, Methods), we obtained 22 cell types (S4 Fig) and visualized the single-cell transcriptomes in a two-dimensional t-distributed stochastic neighbor embedding (tSNE) map [13] (Fig 1C).

**Fig 2:**
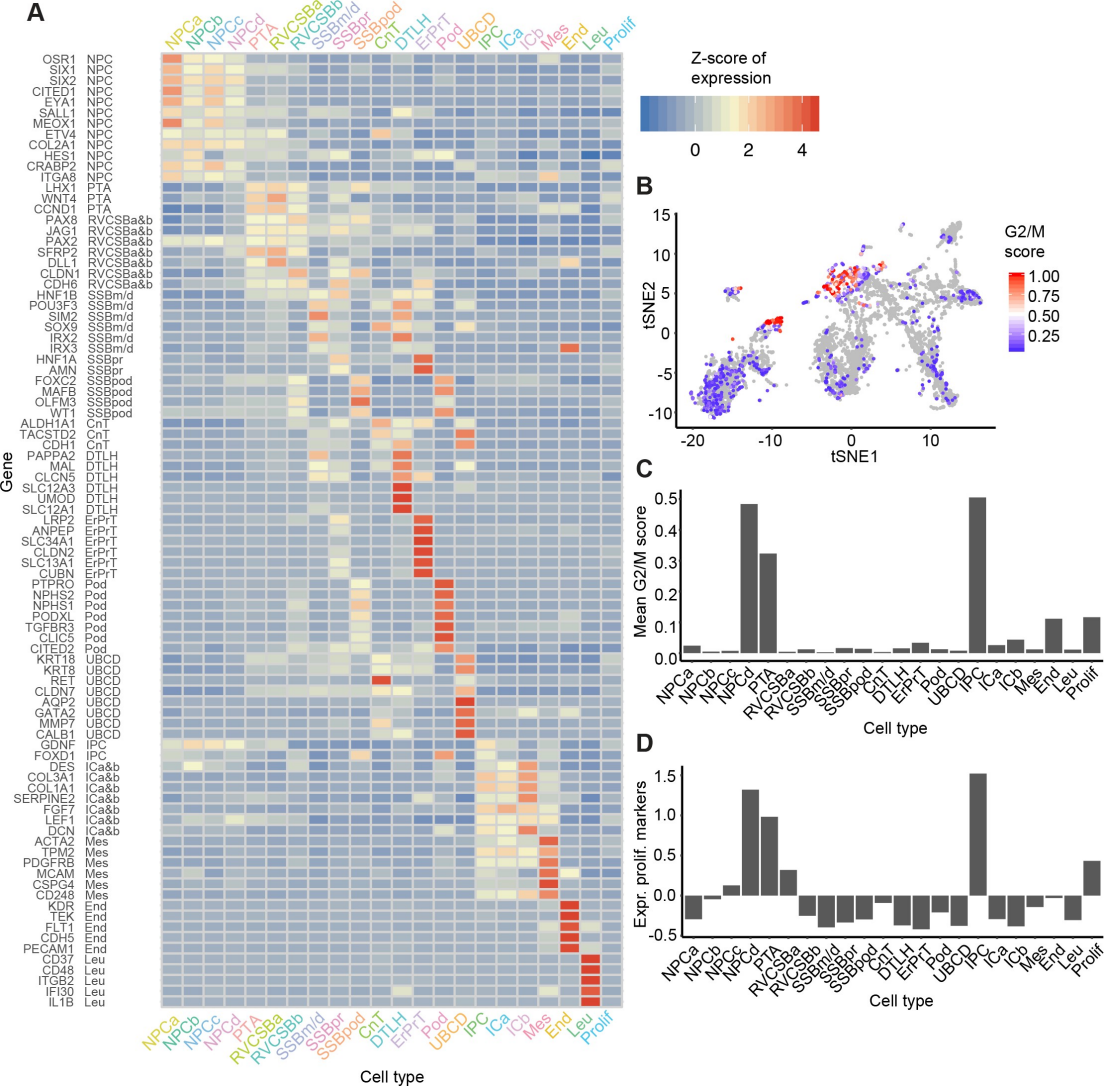
Known markers elucidated the cell types corresponding to each cluster. (A) Heatmap of literature set gene expression in the 22 identified cell types. Expression was Freeman-Tukey transformed, averaged over all cells in a cluster and standardized gene-wise. (B) tSNE map of all cells with color indicating the G2/M score (calculated by the Cyclone tool [15]). This score reflects the likelihood that a cell is in G2/M phase. (C) G2/M scores from panel B averaged over the cells in each cell type. (D) Mean expression of proliferation markers [16] (Z-scores) per cell type. The numerical data underlying this figure can be found in S1 Data. CnT, connecting tubule; DTLH, distal tubule/loop of Henle; End, endothelial cells; ErPrT, early proximal tubule; G2/M, cell cycle phase G2/M; ICa, interstitial cells a; ICb, interstitial cells b; IPC, interstitial progenitor cells; Leu, leukocytes; Mes, mesangial cells; NPCa, nephron progenitor cells a; NPCb, nephron progenitor cells b; NPCc, nephron progenior cells c; NPCd, nephron progenitor cells d; Pod, podocyte; Prolif, proliferating cells; PTA, pretubular aggregate; RVCSBa, renal vesicle/comma-shaped body a; RVCSBb, renal vesicle/comma-shaped body b; scRNAseq, single cell RNA sequencing; SSBm/d, s-shaped body medial/distal; SSBpod, s-shaped body podocyte precursor cells; SSBpr, s-shaped body proximal precursor cells; tSNE, t-distributed stochastic neighbor embedding; UBCD, ureteric bud/collecting duct.

The mean expression levels of the literature set genes showed clear differences between cell types (Fig 2A). NPCs, which were distributed over four distinct clusters (NPCa–d), were marked by the established markers *SIX2* (Fig 1C), *CITED1*, *MEOX1*, and *EYA1*. Expression of these progenitor markers was highest in NPCa, which we hence considered “bona fide” self-renewing NPCs. NPCb showed lower levels of *CITED1* and *SALL1* and higher levels of *GDNF* and *HES1* compared to the other NPC clusters. *HES1*, a transcription factor downstream of NOTCH signaling, is important for further renal cell differentiation. Compared to the other NPC clusters, NPCc showed higher expression of *CRABP2*, which is related to retinoic acid signaling [14]. NPCd exhibited low *OSR1*, *CITED1*, and *MEOX1* expression and increased levels of *LEF1*, a known indicator of NPC induction towards differentiation. Compared to the other NPC subtypes, NPCd were also marked by a larger fraction of cells in G2/M-phase of the cell cycle (Fig 2B and 2C) and a higher expression of proliferation markers (Fig 2D), which indicated faster proliferation. We will discuss the relationship between the various NPC clusters in more detail below (see Heterogeneity in the nephrogenic niche).

Nephrogenesis continues with the creation of the pre-tubular aggregate (PTA) cells, which in turn develops into the RV and CSB. In our data, PTA cells were identified based on high expression of *LHX1* (Fig 1C), *JAG1*, *WNT4*, and *CCND1*. Because RV and CSB are mainly distinguishable by morphology, cells belonging to these two structures were grouped in our analysis (RVCSB). RVCSB cells were marked by the same genes as PTA cells, but they appeared to proliferate less (Fig 2B–2D). Furthermore, they expressed markers reflecting more advanced regional patterning, which allowed us to discriminate between two subtypes (a and b). RVCSBa had a higher expression of genes that were recently associated with the distal RV (*SFRP2*, *DLL1*, *LHX1*), whereas RVCSBb expressed genes that indicate the proximal RV (*CDH6*, *FOXC2*, *MAFB*, *CLDN1*, *WT1*).

The next step in development is the formation of the SSB. In our data set, this structure was represented by three clusters, named according to the part of tubule and glomerular epithelium they are known to give rise to—SSBpr, proximal tubule; SSBm/d, medial/distal; SSBpod, podocytes. SSBpr were identified in our data by markers of the early proximal tubule (ErPrT) (such as *HNF1A*, *CDH6*, *AMN*), as well as low expression of *SLC3A1*, *LRP2*, and *SLC13A1*, which are known to be found in more mature proximal tubule cells. Therefore, SSBpr were likely precursors of the ErPrT cells, which expressed higher levels of early proximal markers together with *CLDN2* (Fig 1C), *ANPEP*, and *SLC34A1*. Another cluster (SSBm/d) accounted for the precursor cells of the loop of Henle and the distal tubule in the SSB. This cluster could be identified by the presence of *IRX1*, *IRX2*, *SIM2*, *SOX9*, *POU3F3*, and *HNF1B*, together with low expression of *PAPPA2* and *MAL* and the absence of *CDH6* and *HNF1A*. Cell types that are known to develop from the SSBm/d were found together in one cluster (distal tubule/loop of Henle [DTLH]). This cluster showed high expression of the distal markers *MAL*, *CLCN5*, *SLC12A3*, and *POU3F3*, which are specific to the distal tubule, as well as *SLC12A1*, *PAPPA2*, and *UMOD*, which are found in the loop of Henle. Finally, cells that likely gave rise to podocytes, SSBpod, clustered separately. These cells showed high expression of *MAFB* and *FOXC2*, both transcription factors necessary for the development of podocyte identity, and low levels of the mature podocytes markers *CLIC5* (Fig 1C), *PTPRO*, *NPHS1*, and *NPHS2*. This cluster also showed the highest expression of *OLFM3*, previously identified as a specific marker of podocyte precursors residing in the visceral part of the proximal segment of the SSB. In contrast to SSBpod, podocytes (Pods) showed higher expression of mature podocyte markers and lower levels of *MAFB*. Differences between SSBpod and Pods will be studied in more detail below (see Podocyte development). Because of the high similarity in gene expression between SSB and capillary loop stage, we could not exclude that the SSB clusters also contained cells from the capillary loop stage.

Cells of the connecting tubule (CnT), which connects the distal tubule to the collecting duct, could also be identified in the data. They shared markers with the collecting duct (such as *ALDH1A1*, *TACSTD2*, and *CDH1*), distal tubule (*SOX9*, *POU3F3*), and UB (*RET*, *KRT8*, *KRT18*, *MMP7*). Cells of the UB and collecting duct (UBCD) were strongly marked by well-known genes like *AQP2* (Fig 1C), *CALB1*, *KRT8*, *KRT18*, *RET*, and *GATA2*, found in the collecting duct as well as the stalk and tip of the UB.

The developing nephrons are surrounded by interstitial tissue, a separate lineage that originates in interstitial progenitor cells (IPCs). We identified IPCs by coexpression of *FOXD1* and *GDNF*. These cells also expressed lower levels of markers known to be found in more mature cells like *PDGFRA* for ICs or *PDGFRB* (Fig 1C) and *ACTA2* for mesangial cells. We identified two subtypes of ICs (a and b), which were similar in their marker gene profile. Compared to IPCs, they lacked *FOXD1* and expressed less (ICa) or no (ICb) *GDNF*. ICa showed high levels of *FGF7*, which has been localized to the renal fibroblasts or stroma surrounding the ureter and the collecting system. ICa also showed high levels of *TPM2* and *ACTA2*, markers of smooth muscle-like cells. ICb, on the other hand, expressed genes like *DCN* (Fig 1C), *DES*, *SERPINE2*, and *COL3A1*, which are known to mark cortical stromal cells. Endothelial cells were identified by markers such as *KDR* and *TEK*, whereas leukocytes showed many specifically expressed genes, such as *CD37* or *CD48*. Finally, one cluster of cells (Prolif) had a higher expression of proliferation markers compared to most other cell types (Fig 2D) but lacked discernible cell type markers.

### Developmental flow

**Fig 3:**
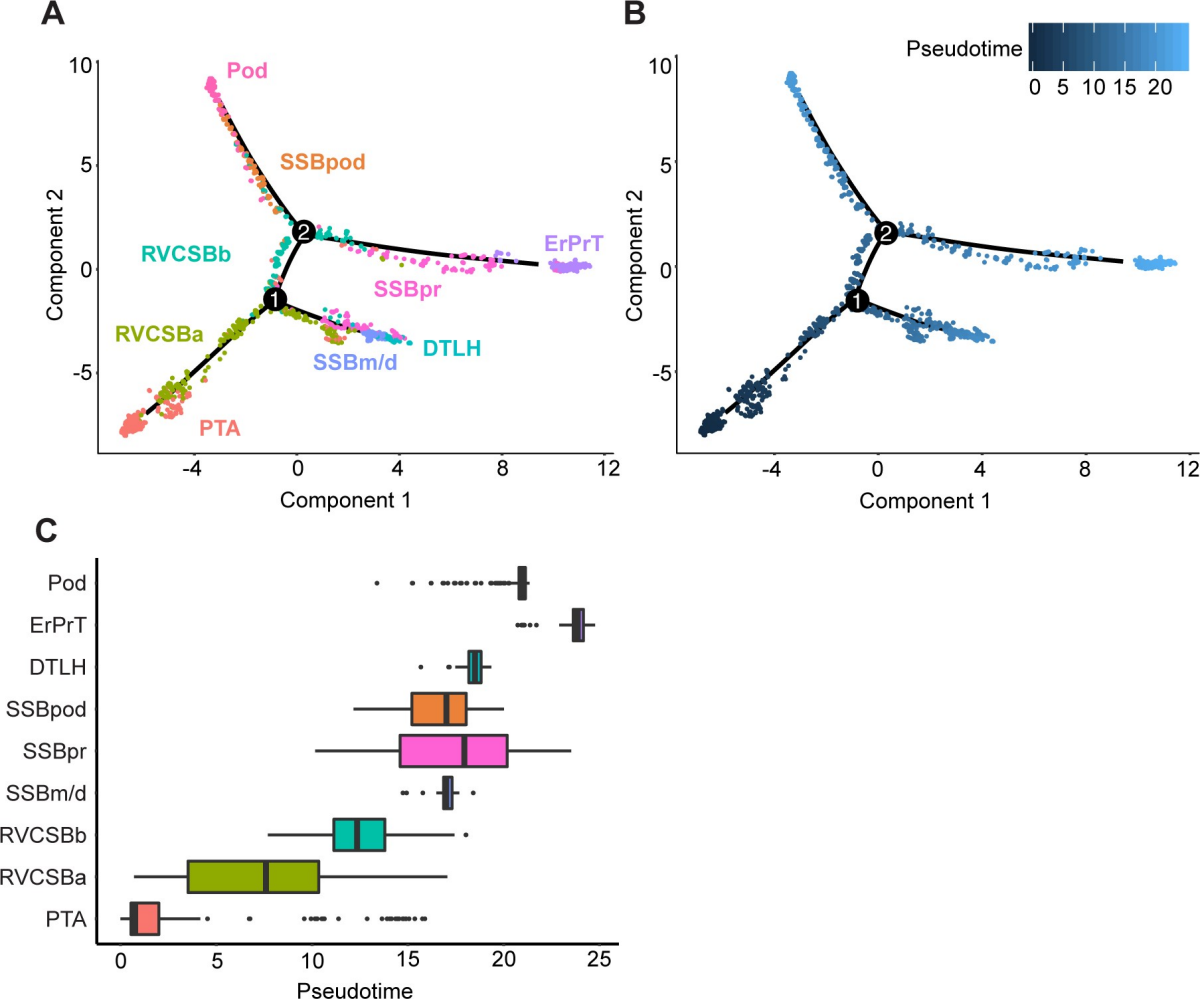
Pseudotime analysis clarified the developmental relationship of the cell clusters. (A) Two-dimensional embedding (with the *DDRTree* algorithm [18]) of all w16 kidney cells, calculated by *Monocle 2* [17]. The graph learned by the algorithm is shown as a black line. Colors and labels indicate cell types. (B) Same embedding as in panel A. Color indicates pseudotime calculated by *Monocle 2*. (C) Box plots of cell type distribution over pseudotime. The numerical data underlying this figure can be found in S1 Data. DTLH, distal tubule/loop of Henle; ErPrT, early proximal tubule; Pod, podocyte; PTA, pretubular aggregate; RVCSBa, renal vesicle/comma-shaped body a; RVCSBb, renal vesicle/comma-shaped body b; SSBm/d, s-shaped body medial/distal; SSBpod, s-shaped body podocyte precursor cells; SSBpr, s-shaped body proximal precursor cells; w16, week 16.

The literature-based analysis of the found clusters seemed to suggest that cells cluster by developmental progression (e.g., NPCs versus PTA cells), as well as location (e.g., RVCSBa, distal, versus RVCSBb, proximal). Because the interpretation of clusters is sometimes based on genes that are expressed in multiple developmental stages, we wanted to retrieve the developmental flow with an independent method. We used *Monocle 2* [17] to learn a graph that represents the developmental hierarchy of the cell types from the PTA on (Fig 3A). Subsequently, cells were placed on a pseudotime scale rooted in the PTA (Fig 3B and 3C). This analysis showed that PTA cells were followed by RVCSBa and RVCSBb, the SSB clusters, and finally the clusters identified as more mature types (DTLH, ErPrT, Pod). Therefore, the clustering was strongly driven by developmental progression. RVCSBa cells were distributed over a fairly broad period of pseudotime and already occurred before branch point 1, which separates proximal from distal cell fates (Fig 3A). This might indicate that some of these cells preceded the RVCSBb, whereas others were primed to develop into distal fates. RVCSBb cells, however, only appeared after branch point 1, which confirmed that they were likely progenitors of proximal cell fates. On three separate branches, SSBm/d preceded DTLH, SSBpr preceded ErPrT, and SSBpod preceded Pods, which confirmed the identity of the SSB clusters. The temporal relationship of the NPC subtypes will be discussed in detail below (see Heterogeneity in the nephrogenic niche).

### Comparison with existing single-cell transcriptomics data

To further confirm the interpretation of the cell clusters, we wanted to compare our data with an existing single-cell transcriptomics study of a w17 fetal kidney by Lindström and colleagues [19]. To that end, we first corrected for batch effects, using a method based on matching mutual nearest neighbors in the two data sets [20]. After correction, the two data sets showed a large degree of overlap (S5A Fig). This allowed us to use the cell types found by Lindström and colleagues to classify the cell clusters found here, using a k-nearest neighbors approach (see Methods). NPCa–c were also classified as NPC by Lindström and colleagues, whereas NPCb were considered “primed NPC,” which supports the notion that NPCb were primed to differentiate. The NPCd cluster was classified as “proliferating cells.” This classification is in agreement with our observation that NPCd seemed to proliferate more than other NPC subtypes (Fig 2B–2D). Because NPCd expressed low levels of NPC markers (such as *SIX2* and *CITED1*), these cells were likely in a transition state between NPCs and PTA cells. Whereas the majority of PTA cells identified here were considered “PTA/RV I” by Lindström and colleagues, RVCSBa cells were spread over multiple cell types. This spread was likely due to the fact that transitory cell types are transcriptionally similar, and their clustering is therefore less robust. Nevertheless, the “PTA/RV II” cluster received most of the RVCSBa cells. RVCSBb cells were called “podocyte precursors” in the Lindström data set, whereas SSBpod as well as Pods were classified as “podocytes.” In our data set, RVCSBb directly preceded SSBpod (Fig 3A), so they could indeed be considered podocyte progenitors. Below, we will show that SSBpod did form a cell state separate from Pods and should not be grouped with them (see Podocyte development). In agreement with our analysis, the majority of SSBpr were classified as “proximal precursor” or “proximal tubule,” and all ErPrT were considered “proximal tubule” by Lindström and colleagues. CnT and DTLH were both classified as “distal/loop of Henle (LOH) precursor.” The fact that two cell types in the study by Lindström and colleagues (“podocytes” and “distal/LOH precursor”) were split in multiple subclusters in our study likely reflects differences in sample preparation. Whereas Lindström and colleagues preferentially released single cells from the nephrogenic niche, here, the whole kidney was used. Consequently, the Lindström data set has a finer resolution of NPCs and early, proliferating cell types, whereas our data set allowed us to resolve more mature cell types. The two data sets therefore complement each other.

### Marker identification

To confirm the inferred cell types and also identify novel markers, we pursued two complementary strategies. First, we determined a set of marker genes based on their usefulness as classifiers for individual cell types: for each gene, the performance of a binary classifier was evaluated by the area under the receiver operating characteristic (AUROC) and combined with expression level filtering (see Methods). This resulted in 88 marker genes (S6A Fig, marker set, S3 Table). Only 11 of these markers overlapped with the 89 genes in the literature set (S6C Fig). To our knowledge, many of the remaining markers had not been associated with kidney development in previous studies. As an independent approach, we used the *KeyGenes* algorithm [21] to identify classifier genes among the 500 most highly variable genes (HVGs), using two-thirds of all cells as a training set. Based on the classifier genes determined by *KeyGenes*, we next predicted the cell types of the remaining one-third of the cells (test set). Cell types could be predicted with an average certainty (id score) of 0.59; 24% of the cells in the test set obtained an id score higher than 0.8. Of the 95 classifier genes (S6B Fig, *KeyGenes set*, S3 Table), 24 were the same as in the marker set, and 14 were common with the literature set (S6C Fig).

Because the interpretation of the found cell clusters was largely based on markers identified in mouse development, we were wondering whether the new markers identified here were informative for the classification of cell types in the mouse kidney. Using a scRNA-seq measurement of cells from a whole P1 mouse kidney [22], we plotted the expression of the newly identified marker genes in single cells (S7 Fig). In many cases, markers that were found to label a particular cell type in the human fetal kidney were coexpressed in the same subset of mouse cells. A few markers, however, were either ubiquitously expressed or almost completely absent. This might be due to interspecies differences.

### Comparison of different developmental ages

**Fig 4:**
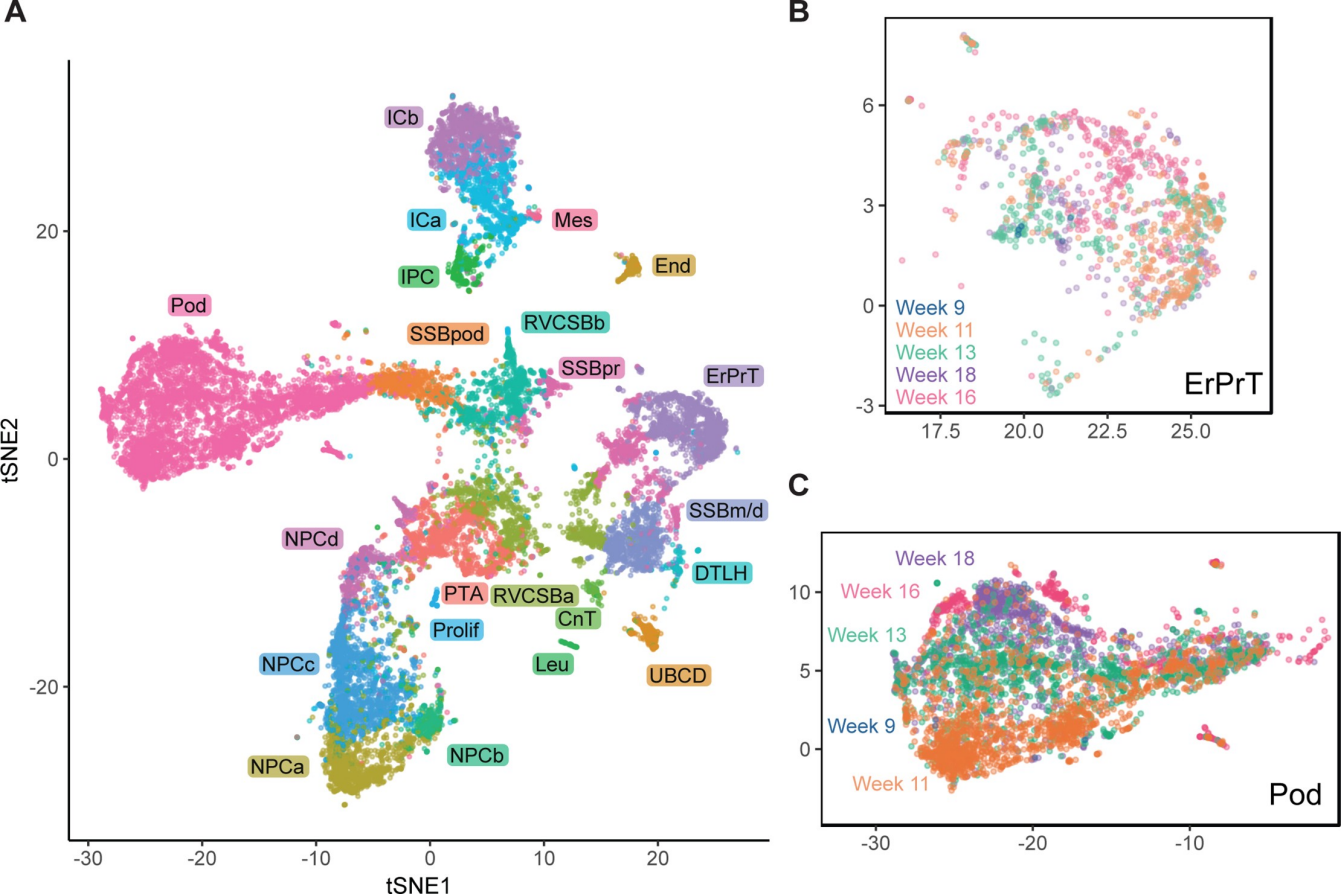
Comparison of different developmental ages suggested continued expression changes in podocytes. (A) tSNE map combining all five samples (w9, w11, w13, w16, w18). Samples were corrected for batch effects by matching mutual nearest neighbors [20]. Cells in the w9, w11, w13, and w18 samples were classified by comparing to the w16 sample using a k-nearest neighbors-based approach (see Methods). (B) tSNE map of all ages restricted to ErPrT. Labels and colors indicate ages. Six outlier cells were omitted from this plot to improve visualization. (C) tSNE map of all ages restricted to Pods. Labels and colors indicate ages. The numerical data underlying this figure can be found in S1 Data. CnT, connecting tubule; DTLH, distal tubule/loop of Henle; End, endothelial cells; ErPrT, early proximal tubule; ICa, interstitial cells a; ICb, interstitial cells b; IPC, interstitial progenitor cell; Leu, leukocyte; Mes, mesangial cells; NPCa, nephron progenitor cells a; NPCb, nephron progenitor cells b; NPCc, nephron progenitor cells c; NPCd, nephron progenitor cells d; Pod, podocyte; Prolif, proliferating cells; PTA, pretubular aggregate; RVCSBa, renal vesicle/comma-shaped body a; RVCSBb, renal vesicle/comma-shaped body b; scRNAseq, single-cell RNA sequencing; SSBm/d, s-shaped body medial/distal; SSBpod, s-shaped body podocyte precursor cells; SSBpr, s-shaped body proximal precursor cells; tSNE, t-distributed stochastic neighbor embedding; tx, transcript; UBCD, ureteric bud/collecting duct; w16, week 16.

By establishing the identity of cell clusters at w16, we obtained a snapshot of cell type diversity in the fetal kidney. To explore whether the identified expression patterns change dynamically throughout development, we analyzed four additional samples from different developmental ages (w9, w11, w13, and w18), which together contained 11,359 usable cells. Using, again, batch correction based on mutual nearest neighbors [20], we visualized all samples in a common tSNE map (Fig 4, S8 Fig). Overall, gene expression in the different samples was largely overlapping for the majority of cell types. For example, proximal tubules cells (ErPrT) appeared at the same positions in the tSNE map in all samples (Fig 4B). The position of Pods, however, shifted systematically across different ages, which corresponds to a continuing change in expression pattern (Fig 4C). This observation might suggest that Pods further matured in terms of their expression pattern after being specified.

Differential expression analysis of Pods of different ages revealed 109 differentially expressed genes (fold change > 2 in any comparison, false discovery rate (FDR) < 0.05, S4 Table). Functional annotation analysis of these genes showed significant enrichment of two gene ontology (GO) terms—“proteinaceous extracellular matrix” (adjusted *p*-value = 1.9 × 10^−3^, including *SPON2*, *BGN*, *COL1A2*, and *CTGF*) and “extracellular exosomes” (adjusted *p*-value = 1.4 × 10^−3^, including *NPNT*, *S100A10*, *ANXA1*, and *EPCAM*). Some of the differentially expressed genes have been shown to be important for kidney development. For example, *NPNT* and *DCN* showed increasing expression from w11 to w18. Knockout of the extracellular matrix protein NPNT in mice decreases the invasion of the UB and causes agenesis or hypoplasia [23]. *NPNT* was further shown to be expressed in the glomerular basement membrane and to be necessary for podocyte adhesion in mice [24]. Ablation of this gene in mice causes podocyte effacement. As in the case of *NPNT*, *DCN* has been reported to be part of the glomerular basement membrane proteins [25]. This gene appeared strongly up-regulated in podocytes between w11 and w13 or w18 (fold changes of 3.25 and 4.6, respectively). The increase of *NPNT* and *DCN* expression over time in our data set could reflect an increase in adhesion between podocytes and glomerular basement membrane. Pods further showed significant differential expressions of genes related to stress, like *HSPA1A* and HSPA1*B* or *NFKB* genes (*NFKB2*, *NFKBIA*, and *REL*), with the highest levels at w18. This might suggest that dissociation-related stress increases with age for podocytes, maybe related to stronger adhesion of the cells, or that stress-related genes have another, physiological role in development.

We would like to emphasize that the observed gene expression changes with age should be considered with caution because they might be related to the differences in genotype between the samples. A much larger number of samples would be necessary to rule out such interindividual differences as a cause.

Having established the identity of the cell clusters, we next wanted to demonstrate how the data set can be used to explore different aspects of kidney development. We specifically focused on the nephrogenic niche, which showed pronounced heterogeneity, and the development of podocytes, which progressed via a distinct, intermediate cell state (SSBpod).

### Heterogeneity in the nephrogenic niche

**Fig 5:**
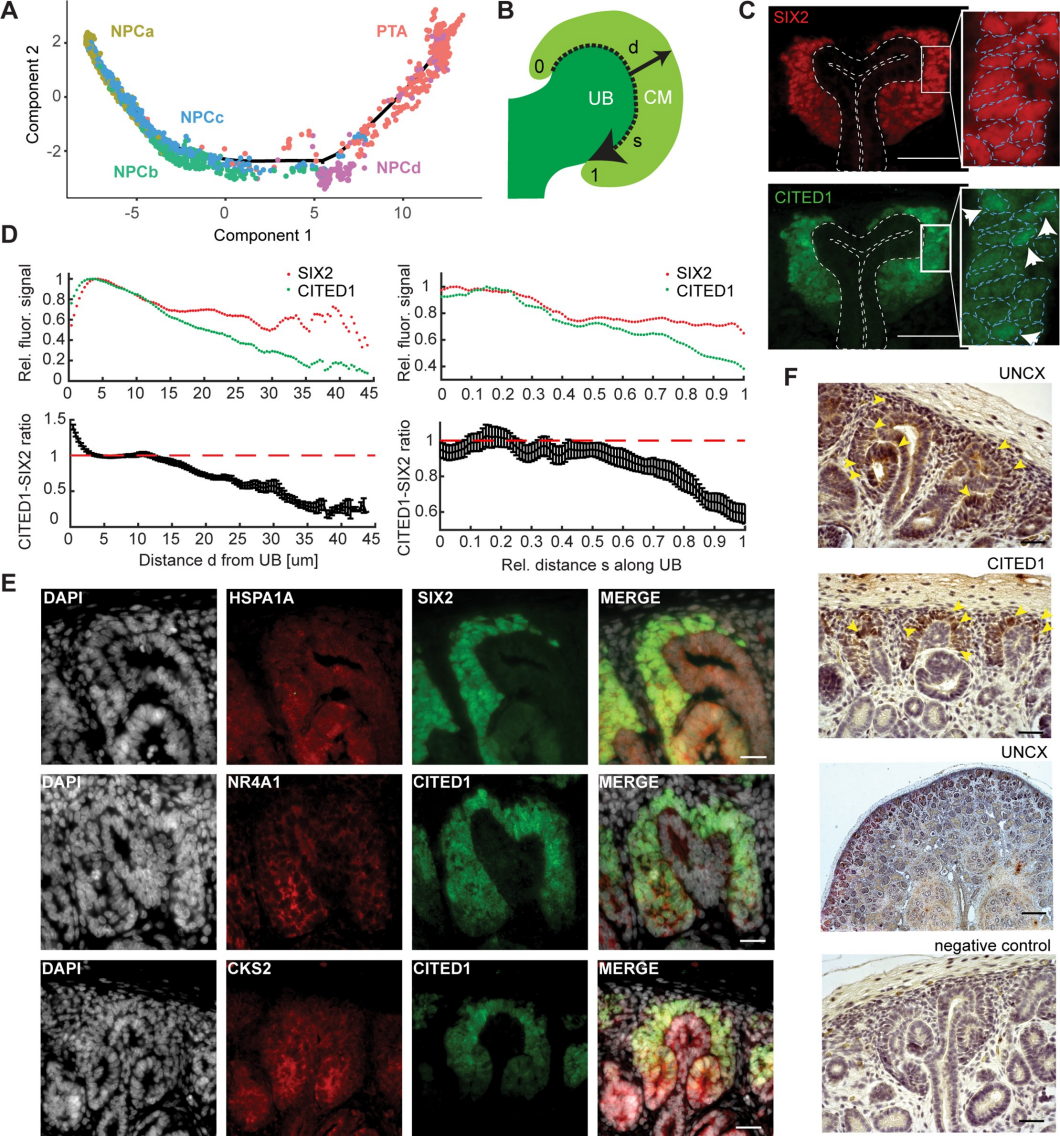
The nephrogenic niche exhibited a complex spatial organization. (A) Pseudotime analysis of the nephrogenic niche (NPC) and the PTA. Two-dimensional *DDRTree* [18] embedding and the learned graph (shown as a black line) were calculated with *Monocle 2* [17]. Labels and colors indicate cell types. (B) Schematic sketch of the CM indicating the distance *d* from the UB to the edge of the CM (solid arrow) and the relative distance *s* along the UB (dashed arrow), in which 0 and 1 represent the top and bottom of the CM, respectively. (C) Representative image of SIX2 and CITED1 immunostaining in a w15 human fetal kidney. Dashed lines in the insets indicate the outline of the nuclei, based on DAPI signal. Arrows in the inset point to cells in which CITED1 is concentrated in the nucleus. Scale bar = 50 μm. (D) Quantification of SIX2 and CITED1 immunostaining with respect to the distance *d* from UB or distance *s* along the UB; see panel A. Error bars indicate the SEM calculated over all evaluated profiles (*n* = 24). (E) Representative image of HSPA1A, NR4A1, and CKS2 immunostaining in a w15 human fetal kidney. Scale bar = 20 μm. (F) Representative image of UNCX and CITED1 immunostaining. Arrowheads indicate the presence of immunostaining signal. Scale bar = 100 μm. The numerical data underlying this figure can be found in S1 Data. CM, cap mesenchyme; NPC, nephron progenitor cell; PTA, pretubular aggregate; SEM, standard error of the mean; UB, ureteric bud; w15, week 15.

The formation of the nephron epithelium starts with the NPCs that differentiate and form the PTA, RV, and CSB. Studies in the mouse suggest that cells in the NPC compartment are not biased towards a particular lineage and patterning is first detectable in the PTA [26]. Nevertheless, the w16 scRNA-seq data indicated the presence of several nephron progenitor subpopulations, NPCa–d. To clarify the temporal relationship of these clusters, we employed *Monocle 2* again to arrange them together with the PTA cells on a pseudotime scale (Fig 5A). NPCa clearly preceded NPCb and c, which seemed to appear around the same pseudotime. NPCd cells followed NPCb and c and preceded PTA. This analysis suggested that NPCa are the “bona fide” NPCs and give rise to NPCb and c. NPCd, which were likely more proliferative than the other NPCs (Fig 2B–2D), seemed to be an intermediate state between (slowly cycling) NPCa–c and the PTA.

To localize the NPC clusters in the tissue, we made use of the fact that they expressed various levels of *CITED1* and *SIX2* (Fig 2A): whereas NPCa and NPCc exhibited roughly similar levels of these markers, NPCb and NPCd had lost *CITED1* almost completely, while retaining some *SIX2* expression. In an immunostaining of a w15 kidney, CITED1 and SIX2 appeared overlapping in a subset of cells (Fig 5B–5D). Quantification of the fluorescence signal (Methods) revealed clear differences between their expression patterns. Whereas SIX2 expression was approximately constant throughout the CM, CITED1 expression decreased, relative to SIX2, with increasing (radial) distance from the UB (Fig 5D). A marked drop of CITED1 was visible between 10 and 20 μm from the UB, which approximately corresponds to the first layer of cells. To exclude that the observed difference between SIX2 and CITED1 expression was due to the different fluorophores on the secondary antibodies, we repeated the experiment with swapped fluorophores. This measurement produced a very similar expression gradient (S9A Fig). To exclude that the observed effect was influenced by PTA found in the CM towards the stalk of the UB, the analysis was repeated, taking only the 20% of CM cells closest to the edge of the cortex into account. A similar expression gradient was observed (S9C Fig). This result implies the existence of a CITED1 low/SIX2 high subpopulation of cells, which are not in contact with the UB. Secondly, we observed that CITED1 decreased relative to SIX2 towards the interface with the PTA and the stalk of the UB (Fig 5D). A similar observation was made when the experiment was repeated with swapped fluorophores (S9B Fig). Taken together, these results suggested that NPCa and NPCc were located closer to the surface of the UB and closer to the tip of the UB compared to the other NPC subtypes. Additionally, we also observed differences in subcellular localization of CITED1 protein within the CITED1 high compartment. Whereas for the majority of cells CITED1 was found in the cytoplasm, in several cells it was concentrated in the nucleus (right inset in Fig 5C). In contrast, SIX2 was always found restricted to the nucleus (left inset in Fig 5C). This observation might indicate that CITED1 was only active in a small population of cells, which would constitute another layer of cell–cell heterogeneity.

In addition to the observed heterogeneity in *CITED1* and *SIX2*, differences between the NPC clusters could also be gleaned from the set of novel markers (marker set, S3 Table).

*TMEM100* and *RSPO3* specifically marked NPCa. *RSPO3* is an activator of the canonical WNT signaling pathway [27], suggesting a role of WNT either in NPC self-renewal or UB branching morphogenesis. Notably, all markers of NPCb (*CACYBP*, *MRPL18*, *ZFAND2A*, *DNAJB1*) were related to the stress response in some form. The markers of NPCc (*CRABP2*, *HAS2*, *MDK*, *HOXC6*), which were also expressed in the other NPC types, are all either targets of retinoic acid or binding it [14,28,29]. *MDK* has been shown to be expressed in the CM of the developing rat kidney, and its neutralization reduced the number of formed nephrons in vitro [30,31]. Finally, the NPCd markers CENPF, HMGB2, CCNB2, and NUSAP1 all have a role in cell cycle regulation or proliferation [16]. HMGB2 was recently implied in the activation of quiescent adult neural stem cells [32].

The observation that markers of NPCb were related to the stress response seemed to suggest that this cluster was created as an artefact of cell dissociation [33], despite our best efforts to remove stressed cells (see Methods). On the other hand, the vast majority of NPCb cells were classified as “primed NPC II” in the Lindström data set (S5 Fig). The fact that NPCb cells were only detected in the w16 and w18 kidneys is consistent with single-cell dissociation becoming increasingly difficult with fetal age, or alternatively, with a progenitor cell aging phenomenon. To explore the differences between NPCb and the other NPC clusters further, we immunostained HSPA1A and NR4A1, both known stress-response genes, in w15 kidney sections (Fig 5E). *HSPA1A* was identified as a marker of NPCb (S9D Fig, S3 Table), whereas *NR4A1* was expressed in multiple NPC clusters but highest in NPCb (S9D Fig). Furthermore, *NR4A1* was also identified in the study by Adam and colleagues [22] to be up-regulated in response to elevated temperatures during enzymatic dissociation. HSPA1A and NR4A1 were both observable in the nephrogenic niche at the level of the stalk of the UB and at the transition to the PTA or RV. Additionally, we studied the expression of *EGR1*, another stress-related gene that marked NPCb, with smFISH (S9E Fig). *EGR1* was mainly found toward the stalk of the UB and in a few cells around the tip of the UB, whereas *SIX2* and *CITED1* transcripts were visible throughout the CM (S9F Fig). Because results obtained in fixed tissue sections are not confounded by dissociation-related artifacts, these immunostainings and smFISH measurements supported the existence of NPCb cells in the fetal kidney.

The fourth NPC cluster, NPCd, was clearly distinguished by proliferation markers (Fig 2B–2D). To locate NPCd in the tissue, we immunostained the cell cycle regulator and NPCd marker *CKS2* (S2G Fig). CKS2 signal could be observed around the stalk of the UB and in RVs (Fig 5E). This result supported the interpretation that NPCd were a proliferating transitory state between NPCa–c and PTA.

Given the crucial role of the nephrogenic niche in the development of nephrons, it is likely that misexpression or mutation of genes that are specifically expressed in NPCs affect kidney function. Mining a database of genome wide association studies (GWAS) revealed that genes that were differentially expressed in NPCs were significantly enriched for association with kidney disease (*p* = 1.7 × 10^−3^, one-sided Fisher’s exact test). No enrichment was found for lung diseases (*p* = 0.21, one-sided Fisher’s exact test) (see Methods, S10 Fig, S4 Table). Unsurprisingly, several of the disease-associated genes are known regulators of kidney development, such as *SALL1*, *SOX11*, and *HAS2* [34–37]. The other identified genes had not been previously associated with kidney development. For example, *DDX1*, which was differentially expressed in NPCs as well as SSBpod, is an RNA helicase that promotes micro-RNA maturation [38]. *UNCX*, which was broadly expressed in all NPC clusters, is a homeobox transcription factor involved in somitogenesis and neurogenesis [39] and has also been found to be up-regulated in the induced mouse nephrogenic mesenchyme in culture [40]. It was recently associated with renal-function–related traits [41] as well as glomerular filtration rate [42–44]. In our data, the expression profile of *UNCX* was similar to that of *CITED1* (S9H Fig). Immunostaining of UNCX confirmed the scRNA-seq results and showed expression of UNCX in the nephrogenic zone, as marked by CITED1 (Fig 5F and S9I Fig). These findings suggested *UNCX* as a novel potential regulator of early nephrogenesis.

### Podocyte development

**Fig 6:**
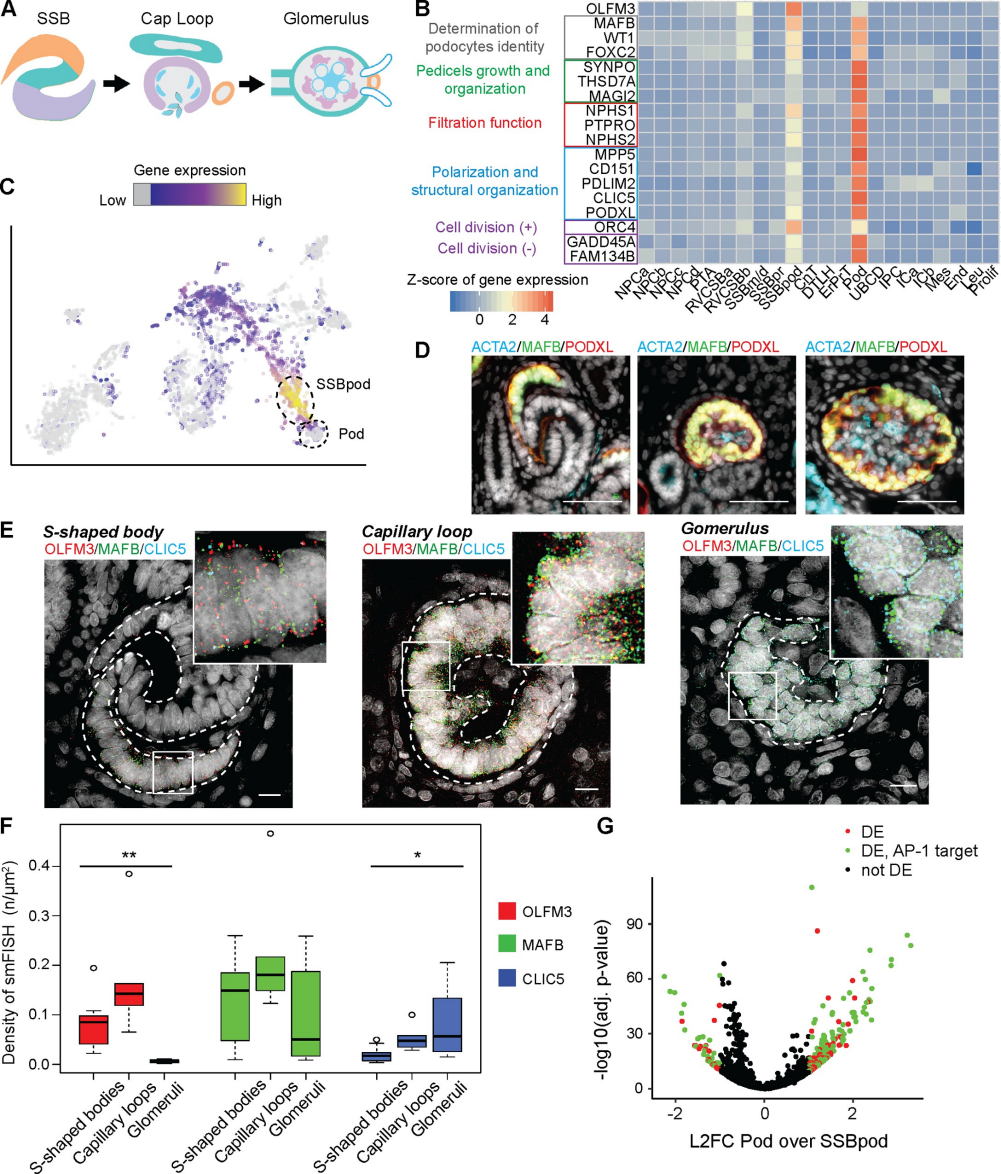
Podocytes developed via a precursor state localized in the visceral proximal SSB. (A) Schematic of development from the SSB to the glomerulus. Regional patterning is shown in color—proximal (purple), medial (green), distal (orange). (B) Expression heat map of the subset of marker set genes that are markers for SSBpod and Pods. Expression was Freeman-Tukey transformed, averaged over all cells in a cluster and standardized gene-wise. (C) Two-dimensional tSNE map showing the expression of *OLFM3*. Expression is indicated by color; expression values of 1 are plotted in gray. (D) Typical images of MAFB, PODXL, and ACTA2 immunostaining in SSB and glomeruli. w15 female kidney. Scale bar = 50 μm. (E) Representative images of smFISH of *OLFM3*, *MAFB*, and *CLIC5* in SSBpod and Pods. w15 female kidney. Scale bar = 10 μm. (F) Box plots of smFISH signal densities in SSB (*n* = 10), capillary loop (*n* = 4), and glomeruli (*n* = 8), for *OLFM3*, *MAFB* and *CLIC5* (* adjusted *p* < 0.05, ** adjusted *p* < 0.0005). w15 female kidney. (G) Volcano plot of differential gene expression between SSBpod and Pod. L2FC Pod over SSBpod versus −log10 (adjusted *p*-value). Genes with an adjusted *p* < 0.05 and L2FC > 1 were considered significant (colored data points). Genes with an AP-1 binding site are shown in red. The numerical data underlying this figure can be found in S1 Data. CnT, connecting tubule; DE, differentially expressed; DTLH, distal tubule/loop of Henle; End, endothelial cells; ErPrT, early proximal tubule; ICa, interstitial cells a; ICb, interstitial cells b; IPC, interstitial progenitor cell; Leu, leukocyte; L2FC, log2 fold change; Mes, mesangial cells; NPCa, nephron progenitor cells a; NPCb, nephron progenitor cells b; NPCc, nephron progenitor cells c; NPCd, nephron progenitor cells d; Pod, podocyte; Prolif, proliferating cells; PTA, pretubular aggregate; RVCSBa, renal vesicle/comma-shaped body a; RVCSBb, renal vesicle/comma-shaped body b; smFISH, single molecule fluorescence in situ hybridization; SSBm/d, s-shaped body medial/distal; SSBpod, s-shaped body podocyte precursor cells; SSBpr, s-shaped body proximal precursor cells; tSNE, t-distributed stochastic neighbor embedding; tx, transcript; UBCD, ureteric bud/collecting duct; w15, week 15.

Another cell type of high relevance for kidney function is the podocyte. This cell type is critical for filtration and is implied in several forms of kidney disease [45]. Podocytes wrap around the glomerular basement membrane (capillary bed) inside Bowman’s capsule (Fig 6A). Clustering (Fig 1C) and pseudotime analysis (Fig 3) of the w16 kidney data set had indicated that development into Pods occurs via a distinct intermediate state that we dubbed SSBpod here. This cell state was likely related or even identical to previously discovered podocyte precursors [19,46]. In the Lindström data set [19], SSBpod and Pods were both classified as “podocytes,” and the RVCSBb were considered “podocyte precursors” (S5B Fig). To show that SSBpod cells were indeed a localizable cell state distinct from Pods, we further investigated their expression pattern (Fig 6B), focusing on known literature markers (literature set) and the marker set (S3 Table). Compared to RVCSBb, SSBpod showed higher expression of *MAFB* and *FOXC2*, which are necessary for the determination of podocyte identity [47,48]. On the other hand, compared to Pods, they exhibited lower expression of genes typically associated with more mature podocytes, like *CLIC5*, *PODXL*, and *PTPRO*. Filtration function-related genes like *NPHS1*, *NPHS2*, and *PTPRO* were expressed at intermediate levels in SSBpod compared to RVCSBb, where they were absent, and Pods, where they are highly expressed. A similar pattern could be observed for genes associated with podocyte polarization or structural organization as well as pedicel growth and patterning. Finally, Pods showed the expression of genes that negatively regulate the cell cycle and support long term survival, consistent with their postmitotic nature [49]. In contrast, SSBpod specifically expressed *ORC4*, which has a function in DNA replication. However, proliferation markers were lowly expressed in both SSBpod and Pods (Fig 2D), which suggested low proliferative potential in both cell types. In contrast to NPCs, association with kidney disease was not significantly enriched among genes differentially expressed in SSBpod (*p*-value = 0.1, one-sided Fisher’s exact test). One of the disease-associated genes was *OLFM3*, which has been associated with glomerular filtration rate [50]. OLFM3, a secreted glycoprotein, has a known function in brain and retina development [51] and has been identified as a marker for podocyte precursors in two independent studies [19,46]. In our data set, it was specifically expressed in SSBpod (Fig 6C) and was a marker for this cell type in the marker set and KeyGenes set (S3 Table).

In order to localize SSBpod, Pods and mesangial cells in situ, we immunostained w15 kidney sections with antibodies for MAFB, PODXL, and ACTA2 (Fig 6D). As expected, PODXL and MAFB were found in Pods at the capillary loop stage and in more mature glomeruli. MAFB staining extended to the proximal segment of the SSB, which indicated that SSBpod may be part of this structure. To locate the SSBpod cells more precisely, we performed smFISH on *CLIC5* and *MAFB*, expressed both in Pods and SSBpod (Fig 6E). We observed a subpopulation of *MAFB*+/*CLIC5−* cells outside the glomeruli, which we identified as the SSBpod. These cells could be found predominantly in the visceral part of the proximal segment of the SSB but also at the capillary loop stage. This result supported the notion that SSBpod were transient cells that preceded (mature) Pods. Having localized the SSBpod, we next wanted to confirm OLFM3 as a marker of this cell type. smFISH of *OLFM3*, *MAFB*, and *CLIC5* showed *OLFM3* to be coexpressed with *MAFB* but absent in cells that were positive for *CLIC5* (Fig 6E), a marker that persists in podocytes in the adult kidney. Quantification of the density of smFISH signals (Fig 6F) showed that *OLFM3* was absent in glomeruli but could be detected in the subpopulation we identified as SSBpod (*MAFB+*/*CLIC5−*). In summary, these results supported *OLFM3* as a robust marker of podocyte precursors.

Finally, we were wondering whether our data set would also allow us to identify candidate mechanisms that drive development from SSBpod to Pods. Differential expression analysis revealed 228 genes that had a significant, bigger than 2-fold changes between SSBpod and Pods (Fig 6G, S4 Table). Among these we found factors belonging to multiple signaling pathways, such as *FGF1*, *VEGFA*, *HES1*, and *EGF1*. *Vegfa* and *Fgf1* are known to have a homeostatic function in podocytes [52–54], whereas *Hes1*, a target of the NOTCH signaling pathway, seems to be necessary for the synthesis of extracellular matrix proteins in these cells [55]. Binding sites for the transcription factor AP-1 were strongly enriched in this set of genes (145 out of 228 genes, adjusted *p*-value = 1.3 × 10^−5^). AP-1 would therefore be an interesting target for perturbation studies in mouse models.

All in all, the results presented here complement other, recent, single-cell transcriptomics studies of the fetal kidney. We demonstrated how the data can be interrogated to find expression patterns that will improve our understanding of human kidney development.

## Discussion

### Heterogeneity in the nephrogenic niche

Building an organ during development requires the careful balance between two fundamental processes—growth and the creation of structure. In many organs, these two functions are reconciled by self-renewing progenitor cells that can be induced to differentiate. In kidney development, NPCs give rise to the epithelium of the nephron, the functional unit of the kidney. To balance growth with patterning, self-renewal and differentiation of NPCs have to be tightly controlled. It is well established that the niche of the NPC plays an important role in this control, but the precise mechanisms are not well understood. In particular, it is not clear how the position and movement of NPCs in the niche might impact the induction towards differentiation.

Heterogeneity in the nephrogenic niche was brought to light first by Mugford and colleagues in 2009 [26] and has been confirmed by multiple recent studies [10,19,46,56,57]. Mugford and colleagues used in situ hybridization to study the localization of transcriptional regulators in E15.5 mouse kidney. Three distinct compartments were defined in the CM—inner capping mesenchyme (which lies closest to the cleft of the UB), outer capping mesenchyme (at the tip of the UB), and induced mesenchyme (at the level of the stalk of the UB). Although all compartments expressed *Six2*, only inner and outer capping mesenchyme expressed *Cited1*. The induced CM was distinguished by Wnt pathway activity, as evidenced by *Wnt4* expression. Several recent scRNA-seq studies confirmed heterogeneity in the nephrogenic niche. Brunskill and colleagues studied an E12.5 mouse kidney and found two subpopulations in the CM, which they classified as uninduced (*Six2* positive, *Cited1* positive) and induced (*Six2* positive, *Cited1* negative) [56]. Among the hundreds of genes that were differentially expressed between these two populations, they found genes related to the Wnt signaling pathway as well as protein vesicular trafficking and degradation. Wang and colleagues also found two subclusters in the CM of the human fetal kidney [57]. They interpreted one subcluster as the self-renewing compartment due to higher expression of markers for cell division. The other subcluster, which showed gene expression related to NOTCH signaling (*HES1*, *HEY1*), was considered induced. Two studies by Lindström and colleagues [10,19] also explored NPC heterogeneity. The first study [10] identified four NPC clusters (self-renewing, primed, differentiating, and proliferating), whereas the second [19] revealed four clusters of NPCs (I–IV), two clusters of primed NPCs (I–II), as well as several clusters of proliferating cells.

In the data set presented here, we identified four clusters of NPCs. Among these, NPCa are most likely the self-renewing compartment. In agreement with the studies by Lindström and colleagues [10,19], they expressed the highest levels of *CITED1* and *TMEM100* compared with the other NPCs. Furthermore, they preceded all other NPC clusters in pseudotime analysis. NPCb showed expression of several genes that modulate NOTCH, bone morphogenetic protein (BMP), and transforming growth factor beta (TGF-beta) pathway activity, as well as low levels of *LEF1*, which has been shown to indicate induction towards differentiation [10,19]. The classification of NPCb as “primed NPC” by comparison to the Lindström data set supported the interpretation of NPCb as a state distinct from NPCa that is primed to differentiate. The fact that we detected NPCb only at w16 and w18 leads us to speculate that NPCb could be the result of continuous changes in the nephrogenic niche over the course of development. The third NPC cluster, NPCc, appeared together with NPCb in pseudotime and was distinguished from the other NPCs by higher expression of genes involved in or regulated by retinoic acid signaling. The retinoic-acid binding protein CRABP2 has been identified as an NPC marker in other reports [10,57]. We speculate that NPCc are the result of spatially varying concentrations of retinoic acid, which is produced in the cortical interstitium [58]. Finally, NPCd appeared between NPCb–c and PTA in pseudotime and were clearly distinguished from the other NPC clusters by increased proliferation, at least as far as that can be inferred from gene expression data. In agreement with our analysis, NPCd were classified as “proliferating cells” by comparison with the Lindström data set [19]. NPCd cells also lowly expressed markers of induction towards differentiation (such as *LEF1*, *LHX1*, *WNT4*), which indicates a transitory state between induced and/or primed NPCs and PTA. The suggested developmental flow from NPCa via NPCb–c to NPCd was supported by a gradual decrease of *OSR1*, which is a well-known marker of the early CM.

By in situ detection of CITED1, SIX2, and other genes, we also explored the spatial localization of the different NPC clusters. NPCa seemed to reside closest to the tip of the UB, the induced and/or primed NPC b and c were situated closer to the stalk, and NPCd were closest to the PTA. This finding is consistent with the recent report of NPCs streaming from their niche at the UB tip towards the UB branch point to form the PTA and RV [19]. On this path, the cells gradually lose the NPC transcriptional program, and differentiation is induced. In the mouse, trajectories of NPCs also seem to have a large stochastic component: NPCs repeatedly detach from the UB and attach again and also shuttle back and forth between the “uninduced region” at the UB tip and the “committed region” around the stalk of the UB [59]. This observation could indicate that varying expression levels of genes such as *CITED1* occur as a consequence of cell migration and are not necessarily functionally relevant. Indeed, *Cited1* knockout has no adverse effects on kidney development in the mouse [60]. Taken together, our results support a model in which (self-renewing) NPCa reside at the tip of the UB, probably in close proximity or even in contact with the UB. Movement away from the UB tip, toward the stalk, is accompanied by decreased *CITED1* expression and transformation to the (induced and/or primed) NPCb–c states. Arrival at the stalk of the UB is characterized by the NPCd expression state, increased proliferation, and eventually transformation to the PTA. It is conceivable that cells sometimes visit the different NPC states in reverse order, which would reconcile this model with the observed high, multidirectional motility of NPCs [59].

### Proximal–distal patterning

In the prevailing model of mammalian kidney development, self-renewing NPCs are not prepatterned to develop into a certain lineage. When the developing nephron first displays signs of proximal–distal patterning is an important, outstanding question. Mugford and colleagues have found evidence that the PTA, which succeeds the NPCs, is already polarized [26]. A recent study by Lindström and colleagues [19] proposed an intriguing mechanism that couples temporal and spatial cues: whereas NPCs that are recruited to the PTA early develop into distal cell types, NPCs that are integrated later contribute to the proximal compartment. In our study, we identified the PTA by known marker genes (*CCND1*, *LHX1*, and *WNT4*) and high proliferation. We were unable to detect any substructure within the PTA, which might be due to the limited resolution of our scRNA-seq method. RV/CSB, the next developmental stage, however, was split in two clusters (a and b). Pseudotime analysis suggested that RVCSBa was a heterogeneous cluster comprising early RV cells (which appeared before RVCSBb) and the distal segment of the RVCSB. This observation is consistent with the time-dependent recruitment model by Lindström and colleagues [19] in the sense that in that model, distal specification precedes proximal patterning.

### Podocyte development

Single-cell transcriptomics studies of various organs have brought to light many new, intermediate cell states. This has provoked the question of whether we should consider gene expression in complex tissues as a continuum rather than a collection of distinct expression profiles. In developmental systems, it is certainly useful to think about gene expression change as a continuous process. Nevertheless, there are clearly distinct intermediate cell states even within linear developmental paths. In our study, we observed that RVCSBb gave rise to Pods via an intermediate state, the SSBpod, which directly preceded the Pods in pseudotime. Specific expression of *OLFM3* made it likely that this cluster is identical to previously identified podocyte precursors, which were marked by this gene [19,46]. Menon and colleagues defined a cluster of “immature” or “early” podocytes, characterized by high *OLFM3* and low *MAFB* expression. In that study, podocytes showed increased *MAFB* expression but loss of *OLFM3* [46]. Lindström and colleagues located *OLFM3* positive cells to the proximal part of the SSB [19]. In our study, we confirmed all of these observations: *OLFM3* was localized to the visceral part of the proximal segment of the SSB, and *OLFM3* negative Pods showed higher expression of mature podocyte markers compared to the *OLFM3* positive SSBpod. Functional annotation analysis of genes that were differentially expressed between SSBpod and Pods revealed enrichment of a binding site for AP-1. This transcription factor has been found to be important for the development of the skin [61], neural precursor cells [62], and the heart valve [63] in mice. A role of AP-1 in kidney development has not been described yet, and further research is needed to elucidate its potential function. The analysis of the kidneys from different gestational ages showed high similarity of cell types across different ages with the exception of Pods. These displayed a systematic change in expression pattern, which might indicate the continued maturation of Pods over time. This observation is in agreement with a study by Brunskill and colleagues [64] in the mouse, which compared embryonic (E13.5 and E15.5) with adult podocytes (defined as *MafB* positive cells). That study found hundreds of genes that were differentially expressed between embryonic and adult podocytes. Furthermore, targeted experiments are needed to demonstrate the possible maturation of podocytes in human kidney development.

In summary, we have leveraged a combination of single-cell transcriptomics and in situ imaging to study the intricate structure of the developing human kidney. The transcriptomics data, accessible via a web application (http://www.semraulab.com/kidney), will be a valuable starting point for discovering gene regulatory mechanisms or finding new disease mechanisms.

## Materials and methods

### Ethics statement

The collection and use of human material in this study was approved by the Medical Ethics Committee from the Leiden University Medical Center (P08.087). The gestational age was determined by ultrasonography, and the tissue was obtained by vacuum aspiration from women undergoing elective abortion. The material from six embryos (w9, male; w11, male; w13, female; w15, female; w16, male; and w18, female) was donated with written informed consent. Questions about the human material should be directed to S. M. Chuva de Sousa Lopes (Lopes@lumc.nl).

### Experimental methods

#### Single-cell dissociation of human fetal kidney

One human embryo of w16 (male) was isolated and the kidney dissected in cold saline solution (0.9% NaCl, Versylene Fresenius, Almere, Netherlands). For sex genotyping, polymerase chain reaction (PCR) for AMELX/Y was used as previously described [65]. The obtained kidney was decapsulated and kept on ice in dissociation buffer (DPBS + Penicillin 100 U/mL + Streptomycin 0.1 mg/mL; all from Life Technologies) before cutting it into 1–2 mm pieces. The pieces were washed three times with washing solution (Advanced DMEM F12 supplemented with ITS commercial solution [Insulin–Transferrin–Selenium; Thermofisher], Glutamax, Penicillin 100 U/mL, and Streptomycin 0.1 mg/mL) with brief centrifugation (160 g) in order to remove as many red blood cells as possible. The washed kidney tissue was then incubated with digestion solution (Trypsin/EDTA solution 0.25% and Collagenase-II 280 U/ml) and incubated overnight at 4°C. The next day, the digestion solution was removed, and the kidney was rinsed with washing solution and incubated with washing solution for 30 min at 37°C with agitation. Subsequently, the sample was sequentially passed through sterile cell strainers of 100, 70, and 40 μm pore size with the help of washing solution. The cells were then centrifuged and counted, and viability was measured to be 78% (trypan blue assay) before proceeding with scRNA sequencing library preparation. Four additional human fetal kidneys (w9, male; w11, male; w13, female; and w18, female) were dissected as described above, but, additionally, live cells were purified by fluorescence activated cell sorting (FACS) before library preparation [66].

#### scRNA-seq library preparation and sequencing

scRNA-seq libraries were prepared using the Chromium Single Cell 3' Reagent Kit, Version 2 Chemistry (10× Genomics) according to the manufacturer's protocol. Libraries were sequenced on a NextSeq500 in Mid Output mode using a version 2, 150-cycle kit (Illumina).

#### Immunostaining

A paraffin-embedded w15 human kidney (female) was sectioned (5 μm) using a RM2255 microtome (Leica Microsystems GmbH) and mounted on StarFrost slides (Waldemar Knittel).

For immunofluorescence, sections were deparaffinized and rehydrated by standard procedures, starting with xylene (twice for 20 min), followed by ethanol with sequential dilution and ending with distilled water, all at room temperature. Antigen retrieval was performed by a double treatment of 10 min in a microwave (97°C) with 0.01 M sodium citrate buffer (pH 6.0). The sample was then allowed to cool down, rinsed three times with PBS, and blocked for 1 h at room temperature in blocking buffer (1% BSA, 0.05% Tween-20 in PBS). Subsequently, sections were incubated overnight with the following antibodies diluted in blocking buffer—rabbit anti-SIX2 (1:100, 11562-1-AP; Proteintech), mouse anti-CITED1 (1:500, H00004435-M03; Novus Biologicals), mouse anti-MAFB (1:200, LS-C336952; LifeSpan Biosciences), rabbit anti-ACTA2 (1:200, ab5694; Abcam), goat anti-PODXL (1:200, AF1658; R&D Systems), and rabbit anti-UNCX (1:10, PA5-69485; Thermo Fisher Scientific), rabbit anti-CKS2 (HPA003424, 1:100; Sigma Aldrich), rabbit anti-NURR77 (NR4A1) (ab13851, 1:50; Abcam Biochemicals), and mouse anti-HSP70 (HSPA1A) (ab2787, 1:50; Abcam Biochemicals). The secondary antibodies were diluted in blocking buffer and applied at room temperature for 1 h followed by nuclear counterstaining with 4′,6-diamidino-2-phenylindole (DAPI; Life Technologies). The secondary antibodies used were Alexa Fluor 647 donkey anti-rabbit (1:500, A-31573; Life Technologies), Alexa Fluor 594 donkey anti-mouse (1:500, A-21203; Life Technologies), and Alexa Fluor 555 donkey anti-goat (1:500, A32727; Life Technologies). The sections were then mounted using ProLong Gold (Life Technologies).

For immunohistochemistry, sections were deparaffinized and blocked as above. After overnight incubation with primary antibodies rabbit anti-UNCX (1:10, PA5-69485; Thermo Fisher Scientific) and mouse anti-CITED1 (1:500, H00004435-M03; Novus Biologicals) in blocking buffer, 0.3% H_2_O_2_ was used to quench endogenous peroxidase activity for 20 min. Next, the sections were incubated with biotin-labeled goat anti-rabbit IgG (1:200, BA-1000; Vector Laboratories) diluted in normal goat serum (1:66, S-1000; Vector Laboratories) or biotin-labeled horse anti-mouse (1:200, BA-2000; Vector Laboratories) diluted in normal horse serum (1:66, S-2000; Vector Laboratories) for 40 min. Sections were then treated for 40 min with avidin-biotin-peroxidase complex (VECTASTAIN Elite ABC HRP Kit, #PK-6100; Vector Laboratories) following the manufacturer’s instructions, followed by DAB (D5637; Sigma-Aldrich) and hematoxylin (1043020025; Merck) and were mounted with Entellan (1079610100; Merck).

#### Single-molecule FISH

Paraffin embedded sections from the w15 human fetal kidney (female) used for immunostaining were also used for smFISH experiments. Paraffin was removed by immersion in xylene twice for 10 min at room temperature. The sections were then rehydrated by sequential immersion in ethanol solutions—100% (2×, 10 min), 85% (2×, 5 min), and 70% (2×, 3 min). Subsequently, sections were permeabilized in 70% ethanol for 5 h before incubation with proteinase-K (P4850; Sigma Aldrich) for 15 min at 37°C (23 μg/mL in TE buffer at pH = 8) and a wash in RNAse-free water (3×, 5 min). smFISH was performed as described previously [67]. Briefly, custom designed smFISH probes (BioCat, S5 Table), labeled with Quasar 570, CAL FLuor Red 610, or Quasar 670, were incubated with the samples for 16 h at 30°C in hybridization buffer (100 mg/mL dextran sulfate, 25% formamide, 2X SSC, 1 mg/mL E.coli tRNA, 1 mM vanadyl ribonucleoside complex, 0.25 mg/mL BSA). Samples were washed twice for 30 min at 30°C with wash buffer (25% formamide, 2X SSC) containing DAPI (1 μg/mL, D9542; Sigma). All solutions were prepared with RNAse-free water. Finally, the sections were mounted using ProlongGold (P36930; Life Technologies) and imaged the next day.

#### Imaging

Immunostained and smFISH-treated kidney sections were imaged on a Nikon Ti-Eclipse epifluorescence microscope equipped with an Andor iXON Ultra 888 EMCCD camera, using a 100× /1.45 Plan Apo Lambda oil objective (Nikon, Tokyo, Japan) and dedicated, custom-made fluorescence filter sets (Nikon). To cover large areas of the sectioned kidney, images of multiple adjacent areas were taken and combined using the tiling feature of the NIS Elements software (Nikon). For imaging of smFISH signals, z-stacks were collected with distances of 0.3–0.5 μm between planes in four fluorescence channels (GFP, Quasar 570, CAL FLuor Red 610, Quasar 670).

### Quantification and statistical analysis

#### scRNA-seq data pruning and normalization

Single-cell expression for the w16 sample was quantified using unique molecular identifiers (UMIs) by 10× Genomics’ “Cell Ranger” software. After removing cells with less than 2,000 transcripts per cell, 8,503 cells were retained for further analysis. On average, 1,789 genes were detected per cell and a median of 4,805 transcripts per cell (S2A Fig). Given the recent report that dissociation can have a significant influence on the single-cell transcriptome [33] and that the kidney is notoriously difficult to dissociate, special attention was paid to dissociation-related artifacts. A group of 1,859 cells with signs of stress were removed from the data set (S2B Fig). These cells had more than 10% of their expression come from mitochondrial genes (*MT-ND1*, *MT-ND2*, *MT-CO1*, *MT-CO2*, *MT-ATP8*, *MT-ATP6*, *MT-CO3*, *MT-ND3*, *MT-ND4L*, *MT-ND4*, *MT-ND5*, *MT-ND6*, *MT-CYB*) or more than 5% from stress markers. Stress markers were defined as those genes that were significantly up-regulated upon prolonged enzymatic incubation of mouse kidney tissue in the study by Adam and colleagues [22] (S2 Table, S2B Fig). Mouse genes from this list were converted to human genes using biomart [68]. Genes of the literature set (S1 Table) only showed small differences between stressed and nonstressed cells (S2C and S2D Fig), and stressed cells did not form a separate cluster (S2E and S2F Fig). Therefore, removing stressed cells did not reduce the cell type diversity in the sample. Additionally, 42 cells had more than 1% of their expression coming from *HBB*, *HBA1*, and *HBA2* and were therefore classified as red blood cells and discarded from any further analysis (S2E Fig). Sporadic expression of hemoglobin genes in other cells was likely due to red blood cells that burst before isolation. The same filtering approach was applied to the samples from the other developmental ages as well as the data from Lindström and colleagues [19]. Raw UMI counts were smoothened by k-nearest neighbors smoothing version 2.1 [12]. This procedure reduces technical noise by sharing information between transcriptionally similar cells, which likely belong to the same cell type. Briefly, the expression profiles of each cell and its k-nearest neighbors were summed (k = 10; distance metric: Euclidean distance of the first 10 PCs with a dither of 0.05). The resulting smoothened count matrix had a higher total count than the original and was therefore scaled back to the original matrix by a global factor. Expression was normalized by the method developed by Lun and colleages [69] (as implemented in the scran [version 1.10.1] R package using the functions quickCluster and computeSumFactors). Normalized gene expression was Freeman-Tukey transformed in further analyses unless stated otherwise.

#### Reduction of dimensionality

Variability of gene expression was calculated using the improvedCV2-function from the scran R package. Intercell distances were calculated using the 5% most HVGs excluding stress markers [22] (S2 Table) and ribosomal genes (obtained from the HGNC website) without any filter for minimum mean expression. For maps of individual samples, we used (1–Pearson correlation) as distance measure. For maps of combined samples, we used Euclidean distance in the MNN-corrected principal component space. All tSNE maps used a perplexity setting of 500. For the DDRTree embedding used with pseudotime analysis, see section Pseudotime analysis.

#### Clustering

Hierarchical cluster analysis was performed using Ward linkage and the same intercell distances as for the reduction of dimensionality. The dendrogram of this clustering was cut at height 0.6 to yield 29 clusters of cells (S3A Fig). The cut off was chosen such that the number of resulting clusters was comparable to the number of cell types expected from the literature on mouse development [70] and other scRNA-seq studies of the human fetal kidney [10,19,46,56,57]. We estimated the number of cell types to be around 20 but created slightly more as a starting point to allow for the discovery of new cell types. On the other hand, we did not want to use a much higher number to avoid overclustering (i.e., creating many clusters that are merely driven by noise, which would then have to be merged manually). The presence of known markers of the different cell types in the kidney (literature set, S1 Table) was then used to identify cell types (S3A Fig). Based on this analysis, some adjacent clusters (clusters 4 and 5, 11 and 12, 15 and 16, and 17 and 18) showed very similar expression of known marker genes of podocytes, ICs, UB, and collecting duct and proximal tubule cells, respectively. In addition, the aforementioned clusters were in close proximity, both in the clustering dendrogram as well as in tSNE space (S3B Fig). Consequently, these clusters were merged. For example, clusters 4 and 5 had similar expression of genes known to be expressed in mature podocytes (*NPHS2*, *PTPRO*, *PODXL*) compared to cluster 6, which showed very weak expression of these genes and had distinctive expression of *OLFM3*, which has been shown to be specifically expressed in podocytes precursors [19,46]. Furthermore, we also merged clusters 7 and 25, which were more distant in the dendrogram of the hierarchical clustering but had very similar literature marker profiles (e.g., *LHX1*, *WNT4*, *CCND1*, *JAG1*, *PAX2*, and *PAX8*) and appeared in close proximity in tSNE space. Finally, clusters 26 and 29 were also merged. Cluster 26 was a heterogeneous cluster of only 56 cells that were spread in tSNE space between multiple other clusters. This cluster was closest to PTA (cluster 29) in terms of literature marker expression (*WNT4*, *LHX1*, and *CCND1*) and differed from it with respect to proliferative state, which may account for the heterogeneous distribution.

#### Combining different data sets

To compare cells from multiple scRNA-seq data sets, we used the fastMNN function [20] implemented in scran (version 1.10.1) on the first 50 principal components of the 5% HVGs without stress markers or ribosomal genes. We used a k-nearest neighbor approach to infer the cell types of unclassified cells from already classified cells. For each unclassified cell, the 20 nearest neighbors in batch-corrected principal component space (Euclidean distance) were determined. The most common cell type among these neighbors was then assigned to the unclassified cell. For the comparison with the data set from Lindström and colleagues [19], we restricted our data set to the nephrogenic niche. The cluster identities for the Lindström and colleagues data set were kindly provided to us by the group of Andrew D. Smith.

#### Cell cycle and proliferation

Cell cycle scores were calculated using the Cyclone tool [15] from the scran (version 1.10.1) R package. A list of proliferation markers was adopted from a publication by Whitfield and colleagues [16].

#### Pseudotime analysis

We used the Monocle 2 algorithm [17] to perform embedding and pseudotime analyses on the 2,594 cells of the nephron epithelium, starting from the PTA (cells classified as PTA, RVCSBa, RVCSBb, SSBm/d, SSBpr, SSBPod, DTLH, ErPrT, or Pods), and separately on the 2,153 cells of the nephrogenic niche (NPC) and the PTA. The 5% HVGs (without stress or ribosomal genes) were used as input to the algorithm. We used the reduceDimension function (max_components = 3 for Fig 3; max_components = 2 for Fig 5A) to run the DDRTree algorithm [18]. The root of the graph learned by DDRTree was placed on the branch that starts with the PTA to obtain the pseudotime shown in Fig 3B.

#### Marker genes and KeyGenes

For each gene, the cluster of interest (COI) was defined as the cluster that had the highest mean expression of the gene. Then, a binary classifier based on an expression threshold was defined: cells with expression above that threshold were considered to be part of the COI. We systematically varied this threshold to create a receiver operating characteristic (ROC) based on the cells’ true cluster identities. The AUROC (area under the ROC) was then used to determine the usefulness of this gene as a marker (rather than the specificity or sensitivity at a specific threshold). We defined genes as marker set candidates if they satisfy all of the following four criteria: (1) they have an AUROC exceeding 0.8, (2) they are detected in at least 80% of the cells in the COI, (3) they have a minimum mean expression of 1.5 in the COI, and (4) they are significantly expressed in at most 25% of the cells outside the COI (S3 Table). Significant expression was defined here as an expression level higher than the 25th percentile of expression in the COI. Subsequently, the top four candidate marker genes per cluster, as ranked by the AUROC, resulted in a final set of 88 marker genes (marker set, S3 Table).

To apply the KeyGenes prediction algorithm [21], two-thirds of the cells were assigned to the training set and one-third to the test set. A multinomial logistic regression model was trained on the training set with LASSO shrinkage using the 500 most HVGs, filtered for stress markers and ribosomal genes. The shrinkage parameter was determined by 20-fold cross validation. To apply the KeyGenes method to single cells, each cell was treated as a sample, and cross validation was used to control for overfitting. The model obtained a list of 95 classifier genes with nonzero weights (KeyGenes set, S3 Table). Thereafter, the cells in the test set were assigned to the cell type with the highest identity (id) score; 84% of the cells in the test set were classified correctly (16% test error). On average, the id score was 0.59, and 24% of the cells in the test set obtained an id score higher than 0.8.

#### Differential expression analysis

For all differential expression analyses, we used EdgeR (version 3.24.0) [71] on raw counts. Normalization and dispersion estimates were calculated by calcNormFactors and estimateDisp, respectively. We modeled gene expression with a negative binomial generalized linear model with glmQLFit. Besides the conditions to be compared, a detection rate for each gene was added to the design matrix. The detection rate is defined as the fraction of cells with nonzero expression. In the comparison of different ages, we excluded the w9 and w16 samples. The w9 sample contained only a few cells, which results in high uncertainty for average gene expression levels. The w16 sample was created separately from the other samples. Therefore, to avoid batch effects, which are not corrected for in the differential expression analysis, we therefore also excluded the w16 sample.

#### GWAS analysis

The NHGRI-EBI GWAS catalog was used to retrieve genes associated with traits related to kidney diseases. Specifically, we selected the following traits: kidney stone, kidney disease, rapid kidney function decline, chronic kidney disease, kidney amyloid deposition measurement, acute kidney injury, type 1 diabetes nephropathy, nephrolithiasis, diabetic nephropathy, proteinuria, GFR change measurement, renal cell carcinoma, serum creatinine measurement, cystatin C measurement, type 2 diabetes nephropathy, immunosuppressive agent, tacrolimus measurement, focal segmental glomerulosclerosis, nephrotic syndrome, membranous glomerulonephritis, lupus nephritis, IgA glomerulonephritis, renal system measurement, and Wegener’s granulomatosis. This selection resulted in a list of 560 genes (S4 Table, Kidney GWAS genes). As a negative control, we also obtained a list of 1,508 genes associated with lung diseases (S4 Table, Lung GWAS genes) by selecting the following traits: lung adenocarcinoma, lung carcinoma, interstitial lung disease, squamous cell lung carcinoma, lung disease severity measurement, family history of lung cancer, non-small cell lung carcinoma, diffusing capacity of the lung for carbon monoxide, pulmonary function measurement, vital capacity, emphysema, idiopathic pulmonary fibrosis, chronic bronchitis, chronic obstructive pulmonary disease, pneumonia, and asthma. We performed a one-sided Fisher’s exact test to determine whether the genes in the GWAS lists were significantly enriched in the genes that were differentially expressed in our clusters of interest.

#### Multiple hypothesis testing

In all cases in which significance is reported, *p*-values were adjusted for multiple hypothesis testing using the Benjamini-Hochberg method.

#### Functional annotation enrichment

To look for enrichment of GO terms or transcription factor binding sites we use the DAVID Functional Annotation tool [72], version 6.8 (https://david.ncifcrf.gov/) with all genes in the human genome as background gene set. For enrichment of transcription factor binding sites, we used the “UCSC_TFBS” category.

#### Image analysis

smFISH image stacks were processed with 3D deconvolution and background correction (rolling ball, radius: 3 pixels = 0.39 μm), using the NIS Elements software (Nikon, Tokyo, Japan). Subsequently, maximum projection was used to create a 2D representation of the image stack. The resulting smFISH images were analyzed with homemade MATLAB scripts. First, autofluorescent background was removed by subtracting the appropriately scaled signal of the GFP channel from each of the other channels. Then a region of interest (ROI) containing the structure of interest was defined manually, and candidate smFISH signals were detected by binarizing the image using a global threshold. Connected components were then counted as smFISH signals if they fulfilled two criteria: their average intensity was bigger than the third quartile of individual pixel intensities and they had an area of three pixels or bigger. The density of smFISH signals in the ROI was calculated as the number of retained connected components divided by the area of the ROI.

Images of immunostaining were pre-processed by background subtraction (rolling ball, radius: 100 pixels = 13 μm) using ImageJ [73]. Quantification of the immunostaining signal was carried out using homemade MATLAB scripts. First, the CM region was segmented manually. Then, cross-sections of the CM, roughly perpendicular to the outline of the UB, were drawn by hand, approximately 30 pixels apart. For the starting point of each cross-section, the contour length *s* along the UB starting from the top of the CM (close to the edge of the cortex) was determined (see Fig 5B). The distance *s* was expressed relative to the total contour length (from top to bottom of the UB). The distance from the UB along the cross-sections was defined to be the distance *d* (see Fig 5B). Fluorescence intensities were then averaged over lines of 30 pixels length perpendicular to the drawn cross-section. The resulting intensity profiles (which depend on *d* and *s*) were then averaged over multiple images and either *s* or *d* to get average intensity profiles depending only on *d* or *s*. Normalization to the maximum intensity of each profile resulted in the intensity profiles reported in Fig 5D. Division of the CITED1 intensity profile by the SIX2 intensity profile gave the ratio plotted in Fig 5D. Accuracy, indicated by error bars in the plots, was quantified as the standard error of the mean calculated over all evaluated profiles.

## Supporting information

S1 Fig HE staining of human fetal kidney tissue revealed characteristic stages of nephrogenesis. Several morphologically distinct stages of nephrogenesis are highlighted by colored lines in images of human fetal kidney sections stained with HE. HE, hematoxylin–eosin.
(TIF)

S2 Fig Removing stressed cells did not bias the scRNA-seq results in the w16 sample. (A) Number of detected genes and total number of transcripts per cell. (B) Relative expression of mitochondrial and stress marker genes per cell. Red line indicates the threshold used to define stressed cells. See Methods for the list of mitochondrial genes and S2 Table for the list of stress markers. (C and D) L2FC and scatter plot of the literature set genes (S1 Table). Red dashed lines indicate fold-change of 0.5 and 2. (E) Principal components one to eight of the top 5% most HVGs for all cells. Blue and red points indicate stressed cells and red blood cells, respectively. Expression values in C–E are normalized to library size and log-transformed with a pseudocount of 1. (F) Fraction of stress markers in the 6,602 remaining cells. tSNE map corresponds to Fig 1C. The numerical data underlying this figure can be found in S1 Data. HVG, highly variable gene; L2FC, log2 fold change scRNA-seq, single-cell RNA sequencing; tSNE, t-distributed stochastic neighbor embedding; w16, week 16.
(TIF)

S3 Fig Adjacent clusters were merged based on similarity in literature set gene expression. (A) Heat map of literature set gene expression. Expression was Freeman-Tukey transformed averaged over all cells in the 29 clusters found by hierarchical clustering (indicated by the dendrogram on top of the heat map) and standardized gene-wise. Cluster average cell cycle scores, calculated by Cyclone [15] as well as average expression of proliferation markers [16], are indicated by colored circles below each cluster (Z-score of the mean score or mean expression). (B) tSNE maps highlighting the clusters that were merged to give the cell types indicated in the titles of each map. (Inset lower right) Table listing the numbers of cells in each of the 29 original clusters. The numerical data underlying this figure can be found in S1 Data. tSNE, t-distributed stochastic neighbor embedding.
(TIF)

S4 Fig Most HVGs adequately described all cell clusters. (A) Heat map of 2,034 randomly chosen cells (maximum 100 per cluster) and the five most HVGs with a minimum mean expression of 0.01 excluding stress markers (S2 Table) and ribosomal genes. Genes were assigned to clusters based on highest mean expression within that cluster. Values shown are the ranks of nonzero cells (cells with no expression receive rank 0) divided by the highest rank per gene. The numerical data underlying this figure can be found in S1 Data. HVG, highly variable gene.
(TIF)

S5 Fig Comparison with an existing single-cell transcriptomics data set showed congruent expression profiles despite differences in cell type distribution. (A) Two-dimensional tSNE maps comparing the data presented here with the data from Lindström and colleagues [19] both restricted to the nephrogenic niche by their own classification. The map was calculated using both data sets after batch correction [20]. (Top) Only cells measured in this study are shown. Color and labels indicate the classification developed in this study. (Middle) Same tSNE map as above. Color indicates the data set. (Bottom) Same tSNE map as above. Only cells measured by Lindström and colleagues are shown. Color and labels indicate the classification by Lindström and colleagues. (B) Confusion matrix relating the cells measured in this study to the classification by Lindström and colleagues. After batch correction, cells measured here were mapped on the cells in the Lindström and colleagues data set using a nearest neighbors-based approach (see Methods). The numerical data underlying this figure can be found in S1 Data. tSNE, t-distributed stochastic neighbor embedding.
(TIF)

S6 Fig An ROC-based method and KeyGenes-identified novel marker genes. (A) Expression heat map of the 88 genes identified by a method that evaluates the ROC for each gene (marker set, S3 Table). Expression was Freeman-Tukey transformed, averaged over all cells in a cluster, and standardized gene-wise. (B) Expression heat map of the 95 genes identified by the KeyGenes algorithm (KeyGenes set, S3 Table). Expression was Freeman-Tukey transformed, averaged over all cells in a cluster, and standardized gene-wise. (C) Euler diagram of the literature set, marker set, and KeyGenes set (S3 Table). The numerical data underlying this figure can be found in S1 Data. ROC, receiver operating characteristic.
(TIF)

S7 Fig Newly defined markers clustered cells in the P1 mouse kidney. Expression of the marker genes identified in this study (marker set, S3 Table) in single-cell transcriptomics data of a P1 mouse kidney [22]. Cells were associated with cell types by considering the six marker genes with the highest standardized expression. For each cell, the cell type with the highest representation among this set of six genes was then associated with the cell. The numerical data underlying this figure can be found in S1 Data.
(TIF)

S8 Fig Samples of different developmental ages have a similar cell type diversity. tSNE map calculated for all five samples (w9, w11, w13, w16, w18) combined after batch correction [20]. Developmental age is indicated by color. The numerical data underlying this figure can be found in S1 Data. tSNE, t-distributed stochastic neighbor embedding; w, week.
(TIF)

S9 Fig The nephrogenic niche was heterogeneous. (A and B) Quantification of SIX2 and CITED1 immunostaining with respect to the distance *d* from UB or distance *s* along the UB. Compared to the data shown in Fig 5, the fluorophores on the secondary antibodies were swapped. Error bars indicate the SEM calculated over all evaluated profiles (*n* = 19). (C) Quantification of SIX2 and CITED1 immunostaining with respect to the distance *d* from UB in which only cells with a relative distance *s* (along the UB) < 0.2 were taken into account. Error bars indicate the SEM calculated over all evaluated profiles (*n* = 19). (D) tSNE map showing expression of *HSPA1A* and *NR4A1*. Expression is indicated by color; expression values of 1 are plotted in gray. (E) tSNE map showing expression of *EGR1*. Expression is indicated by color; expression values of 1 are plotted in gray. (F) smFISH of *SIX2*, *CITED1*, and *EGR1*. The three insets at the bottom correspond to the three areas marked by solid boxes in the main image. Scale bar = 25 μm. (G) tSNE map showing expression of *CKS2*. Expression is indicated by color; expression values of 1 are plotted in gray. (H) tSNE maps showing expression of *CITED1* and *UNCX*. Expression is indicated by color; expression values of 1 are plotted in gray. (I) Immunostaining of CITED1 and UNCX. Scale bar = 10 μm. The numerical data underlying this figure can be found in S1 Data. SEM, standard error of the mean; smFISH, single molecule fluorescence in situ hybridization; tSNE, t-distributed stochastic neighbor embedding; UB, ureteric bud.
(TIF)

S10 Fig Disease-associated genes were specifically expressed in transient cell types. Expression of genes from GWAS traits related to kidney disease. Disease phenotypes associated with these genes are indicated by color; genes were filtered for high expression in cluster(s) of interest relative to all other cell types. Expression was Freeman-Tukey transformed, averaged over all cells in a cluster, and standardized gene-wise. (A) Disease-associated genes expressed in early nephron progenitor states (NPC to PTA). (B) Disease-associated genes expressed in SSBpod. The numerical data underlying this figure can be found in S1 Data. GWAS, genome-wide association studies; NPC, nephron progenitor cell; PTA, pretubular aggregate; SSBpod, s-shaped body podocyte progenitor.
(TIF)

S1 Table Literature marker set and references. (XLS)

S2 Table Stress markers. (XLS)

S3 Table New marker sets. (XLS)

S4 Table Differential expression analysis. (XLS)

S5 Table Probes used for smFISH. smFISH, single molecule fluorescence in situ hybridization.
(XLS)

S1 Data Numerical data for all figures. (XLS)

## Abbreviations

AUROC: area under the ROC
BMP: bone morphogenetic protein
CM: cap mesenchyme
CnT: connecting tubule
COI: cluster of interest
CSB: comma-shaped body
DTLH: distal tubule/loop of Henle
End: endothelial cells
ErPrT: early proximal tubule
FDR: false discovery rate
GDNF: glial cell-derived neurotrophic factor
GO: gene ontology
GWAS: genome-wide association studies
HVG: highly variable genes
IC: interstitial cells
IPC: interstitial progenitor cells
id: identity
Leu: leukocytes
LOH: loop of Henle
Mes: mesangial cells
MM: metanephric mesenchyme
NPC: nephron progenitor cells
PCR: polymerase chain reaction
Pod: podocyte
PTA: pretubular aggregate
Prolif: proliferating cells
RV: renal vesicle
RET: ret proto-oncogene
ROC: receiver operating characteristic
ROI: region of interest
RVCSB: renal vesicle/comma-shaped body
scRNA-seq: single-cell RNA sequencing
SSB: s-shaped body, smFISH, single-molecule fluorescence in situ hybridization
SSBm/d: s-shaped body medial/distal
SSBpod: s-shaped body podocyte precursor cells
SSBpr: s-shaped body proximal precursor cells
TGF: transforming growth factor
tSNE: t-distributed stochastic neighbor embedding
UB: ureteric bud
UBCD: ureteric bud/collecting duct
w16: week 16
