# Supplementary figures and images for: Single-cell transcriptomics reveals gene expression dynamics of human fetal kidney development
Source: PLoS Biol. 2019 Feb 21;17(2):e3000152. doi: 10.1371/journal.pbio.3000152 (PMC6400406; doi:10.1371/journal.pbio.3000152)

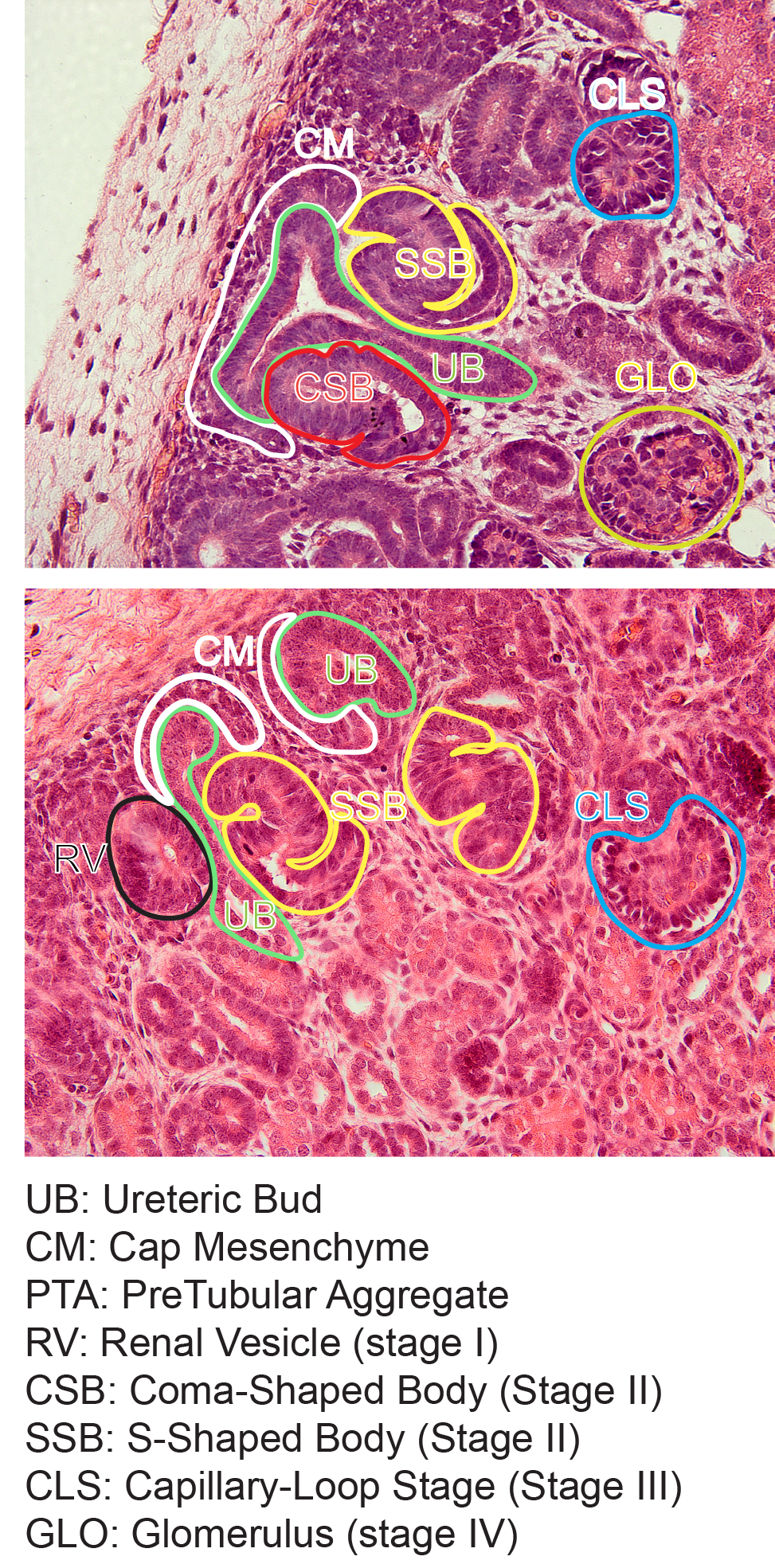

Supplement: S1 Fig — Several morphologically distinct stages of nephrogenesis are highlighted by colored lines in images of human fetal kidney sections stained with HE. HE, hematoxylin–eosin. (TIF) [file pbio.3000152.s001.tif]

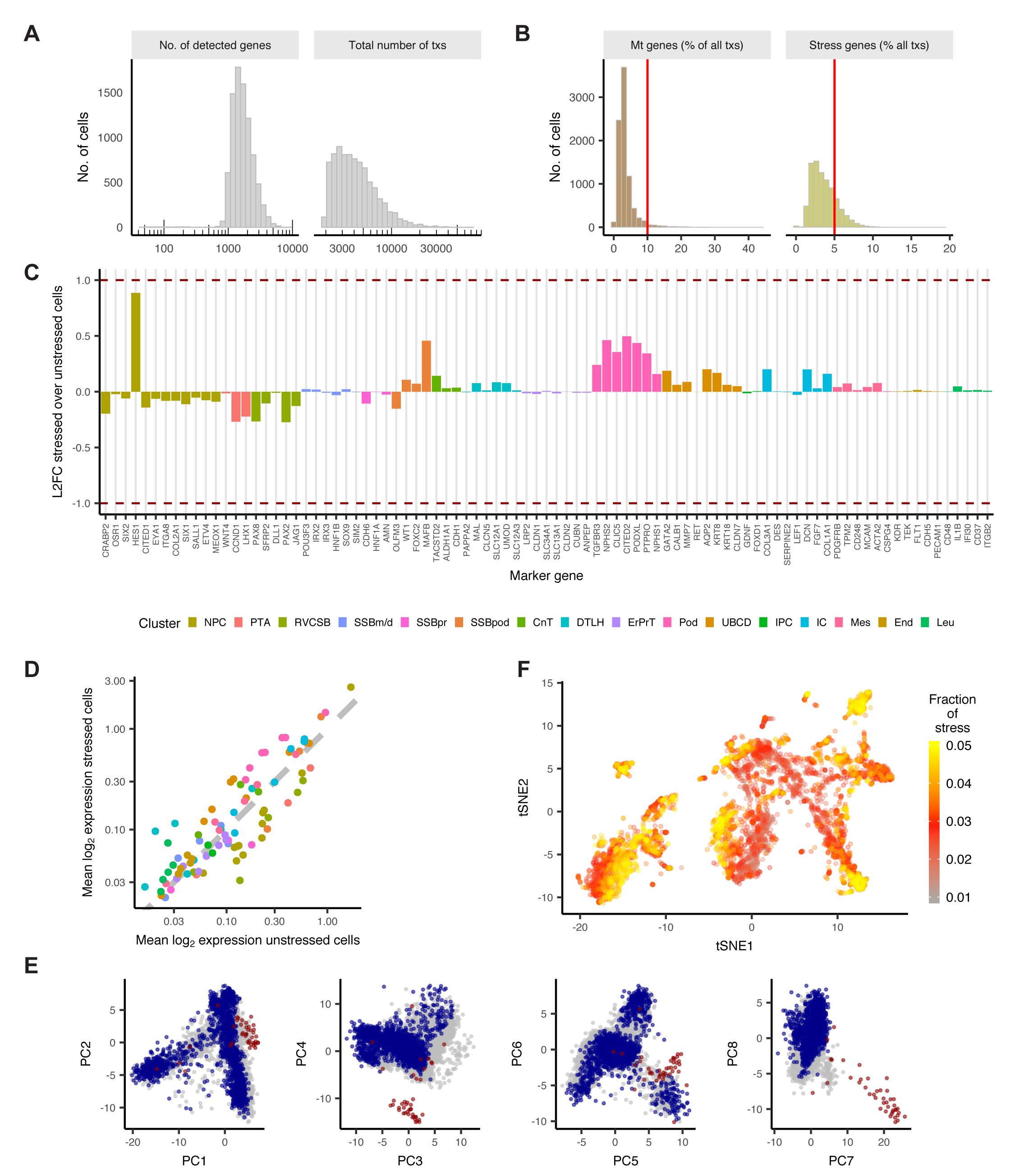

Supplement: S2 Fig — (A) Number of detected genes and total number of transcripts per cell. (B) Relative expression of mitochondrial and stress marker genes per cell. Red line indicates the threshold used to define stressed cells. See Methods for the list of mitochondrial genes and S2 Table for the list of stress markers. (C and D) L2FC and scatter plot of the literature set genes (S1 Table). Red dashed lines indicate fold-change of 0.5 and 2. (E) Principal components one to eight of the top 5% most HVGs for all cells. Blue and red points indicate stressed cells and red blood cells, respectively. Expression values in C–E are normalized to library size and log-transformed with a pseudocount of 1. (F) Fraction of stress markers in the 6,602 remaining cells. tSNE map corresponds to Fig 1C. The numerical data underlying this figure can be found in S1 Data. HVG, highly variable gene; L2FC, log2 fold change scRNA-seq, single-cell RNA sequencing; tSNE, t-distributed stochastic neighbor embedding; w16, week 16. (TIF) [file pbio.3000152.s002.tif]

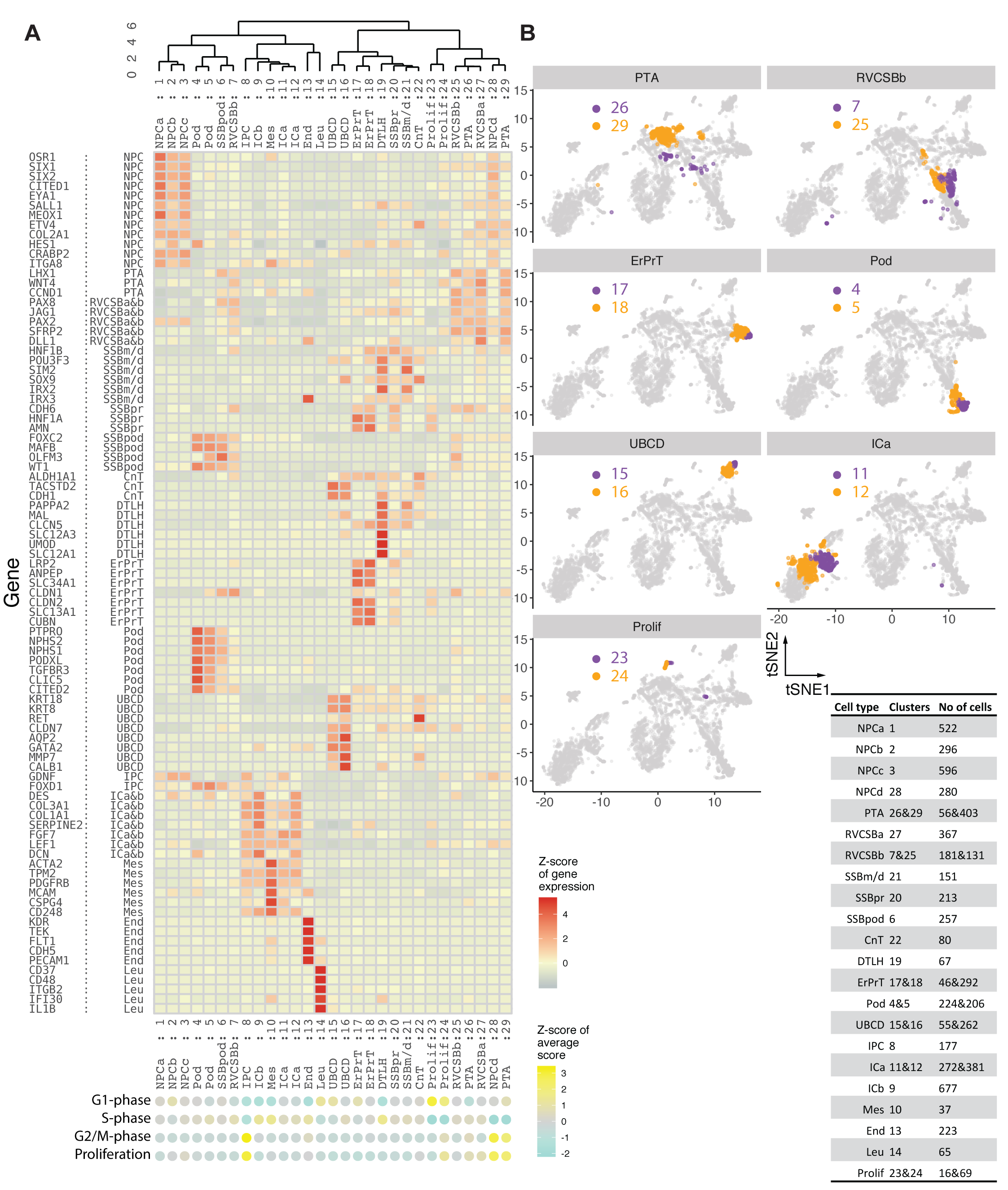

Supplement: S3 Fig — (A) Heat map of literature set gene expression. Expression was Freeman-Tukey transformed averaged over all cells in the 29 clusters found by hierarchical clustering (indicated by the dendrogram on top of the heat map) and standardized gene-wise. Cluster average cell cycle scores, calculated by Cyclone [15] as well as average expression of proliferation markers [16], are indicated by colored circles below each cluster (Z-score of the mean score or mean expression). (B) tSNE maps highlighting the clusters that were merged to give the cell types indicated in the titles of each map. (Inset lower right) Table listing the numbers of cells in each of the 29 original clusters. The numerical data underlying this figure can be found in S1 Data. tSNE, t-distributed stochastic neighbor embedding. (TIF) [file pbio.3000152.s003.tif]

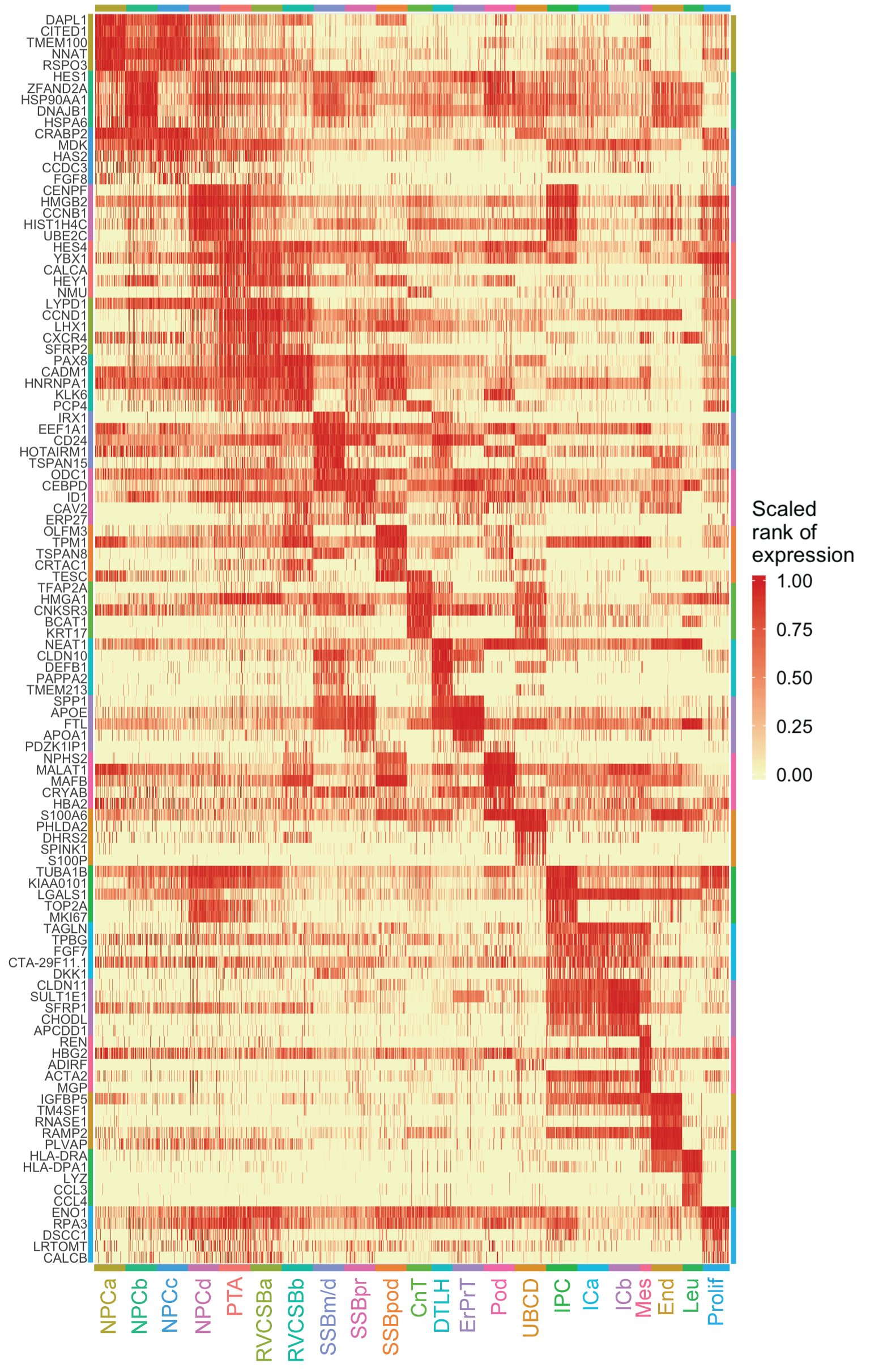

Supplement: S4 Fig — (A) Heat map of 2,034 randomly chosen cells (maximum 100 per cluster) and the five most HVGs with a minimum mean expression of 0.01 excluding stress markers (S2 Table) and ribosomal genes. Genes were assigned to clusters based on highest mean expression within that cluster. Values shown are the ranks of nonzero cells (cells with no expression receive rank 0) divided by the highest rank per gene. The numerical data underlying this figure can be found in S1 Data. HVG, highly variable gene. (TIF) [file pbio.3000152.s004.tif]

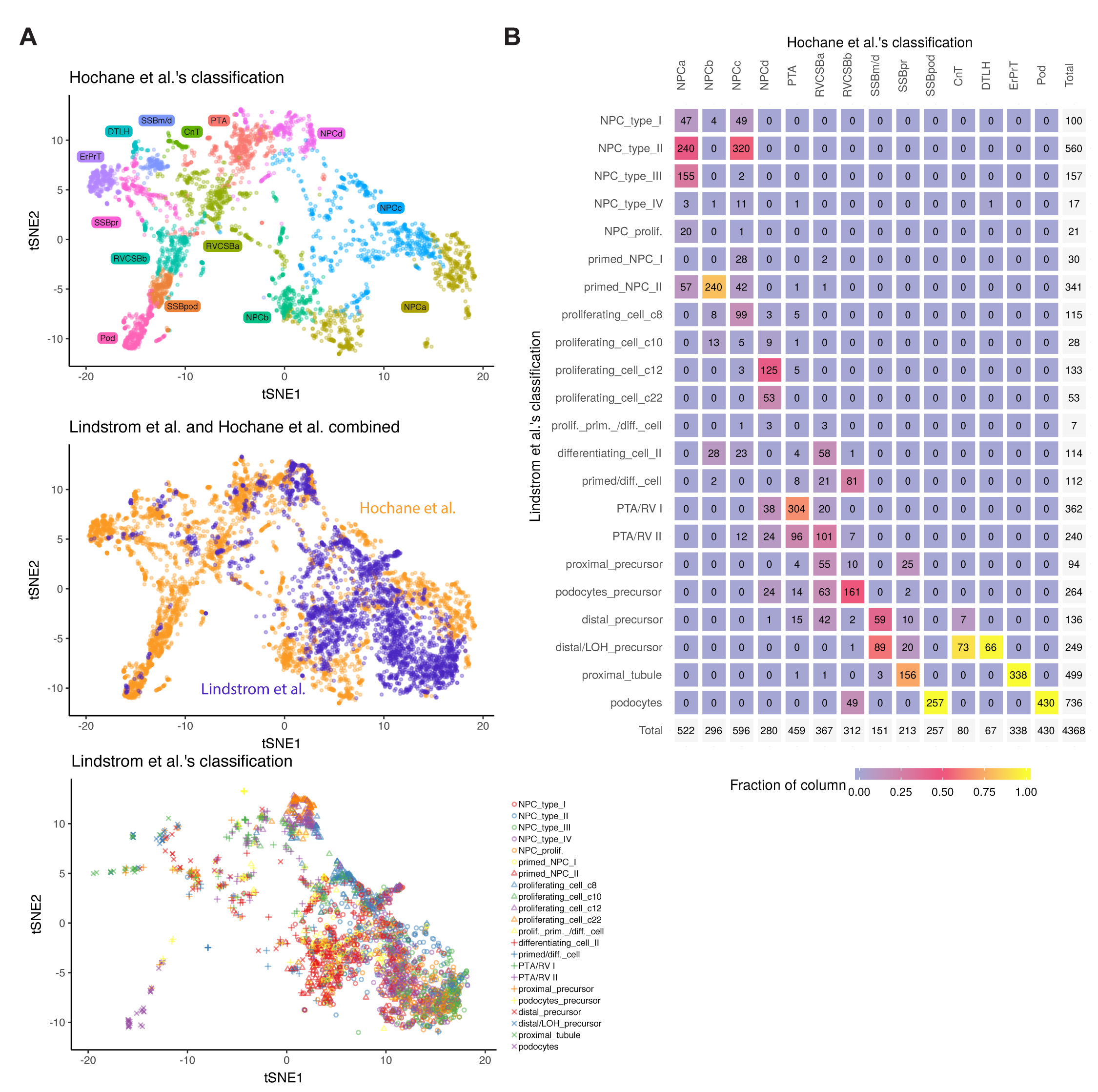

Supplement: S5 Fig — (A) Two-dimensional tSNE maps comparing the data presented here with the data from Lindström and colleagues [19] both restricted to the nephrogenic niche by their own classification. The map was calculated using both data sets after batch correction [20]. (Top) Only cells measured in this study are shown. Color and labels indicate the classification developed in this study. (Middle) Same tSNE map as above. Color indicates the data set. (Bottom) Same tSNE map as above. Only cells measured by Lindström and colleagues are shown. Color and labels indicate the classification by Lindström and colleagues. (B) Confusion matrix relating the cells measured in this study to the classification by Lindström and colleagues. After batch correction, cells measured here were mapped on the cells in the Lindström and colleagues data set using a nearest neighbors-based approach (see Methods). The numerical data underlying this figure can be found in S1 Data. tSNE, t-distributed stochastic neighbor embedding. (TIF) [file pbio.3000152.s005.tif]

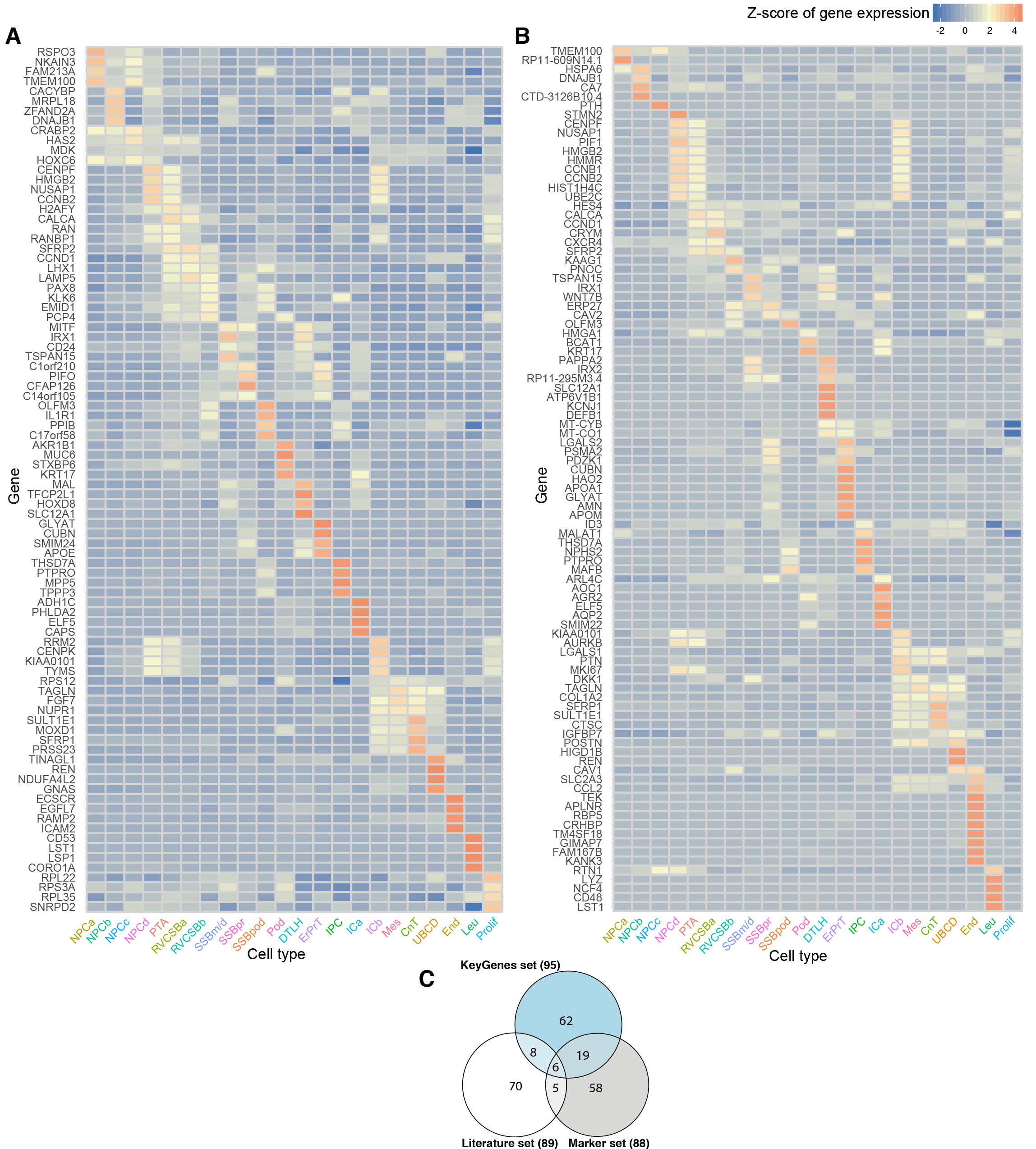

Supplement: S6 Fig — (A) Expression heat map of the 88 genes identified by a method that evaluates the ROC for each gene (marker set, S3 Table). Expression was Freeman-Tukey transformed, averaged over all cells in a cluster, and standardized gene-wise. (B) Expression heat map of the 95 genes identified by the KeyGenes algorithm (KeyGenes set, S3 Table). Expression was Freeman-Tukey transformed, averaged over all cells in a cluster, and standardized gene-wise. (C) Euler diagram of the literature set, marker set, and KeyGenes set (S3 Table). The numerical data underlying this figure can be found in S1 Data. ROC, receiver operating characteristic. (TIF) [file pbio.3000152.s006.tif]

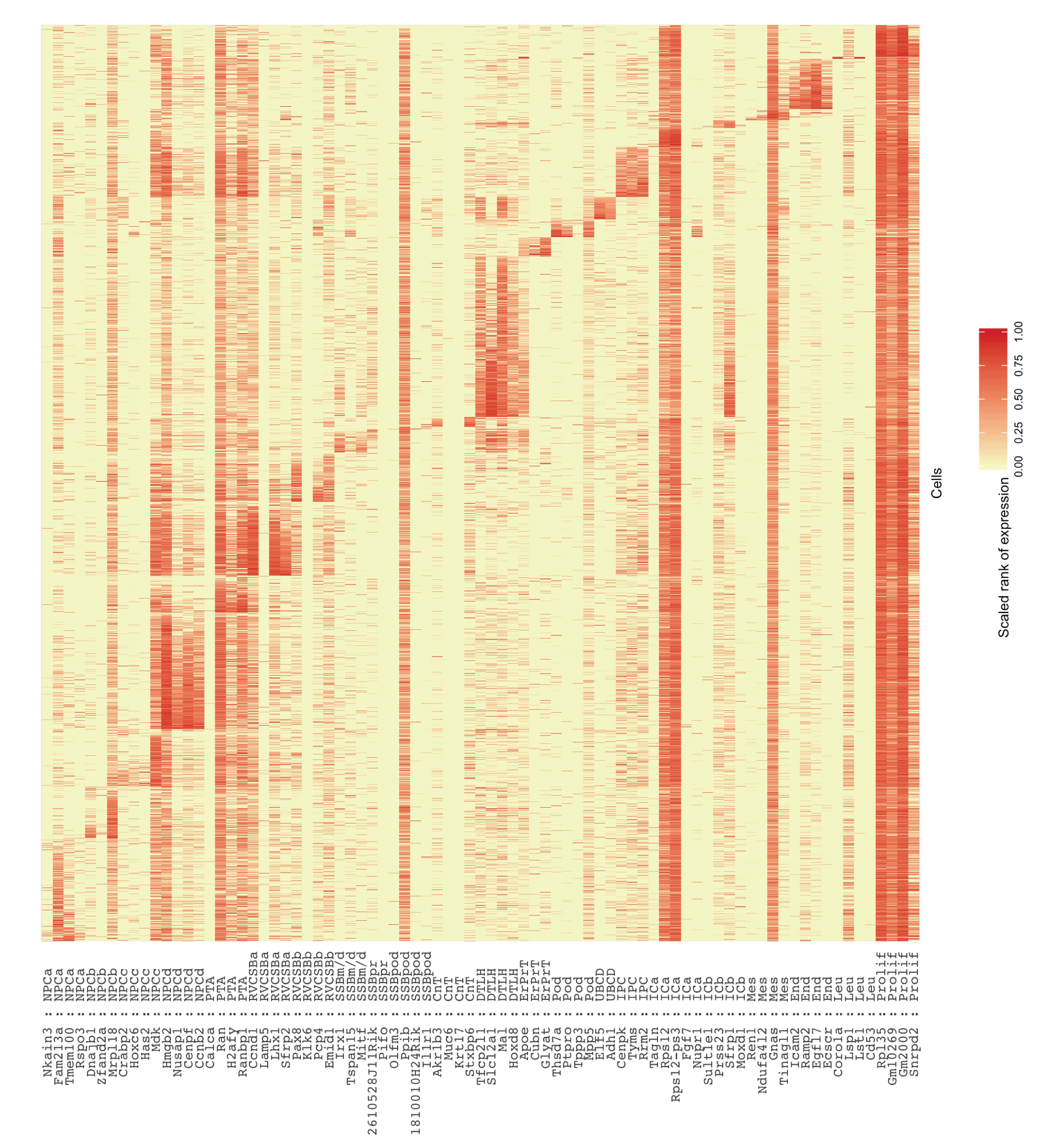

Supplement: S7 Fig — Expression of the marker genes identified in this study (marker set, S3 Table) in single-cell transcriptomics data of a P1 mouse kidney [22]. Cells were associated with cell types by considering the six marker genes with the highest standardized expression. For each cell, the cell type with the highest representation among this set of six genes was then associated with the cell. The numerical data underlying this figure can be found in S1 Data. (TIF) [file pbio.3000152.s007.tif]

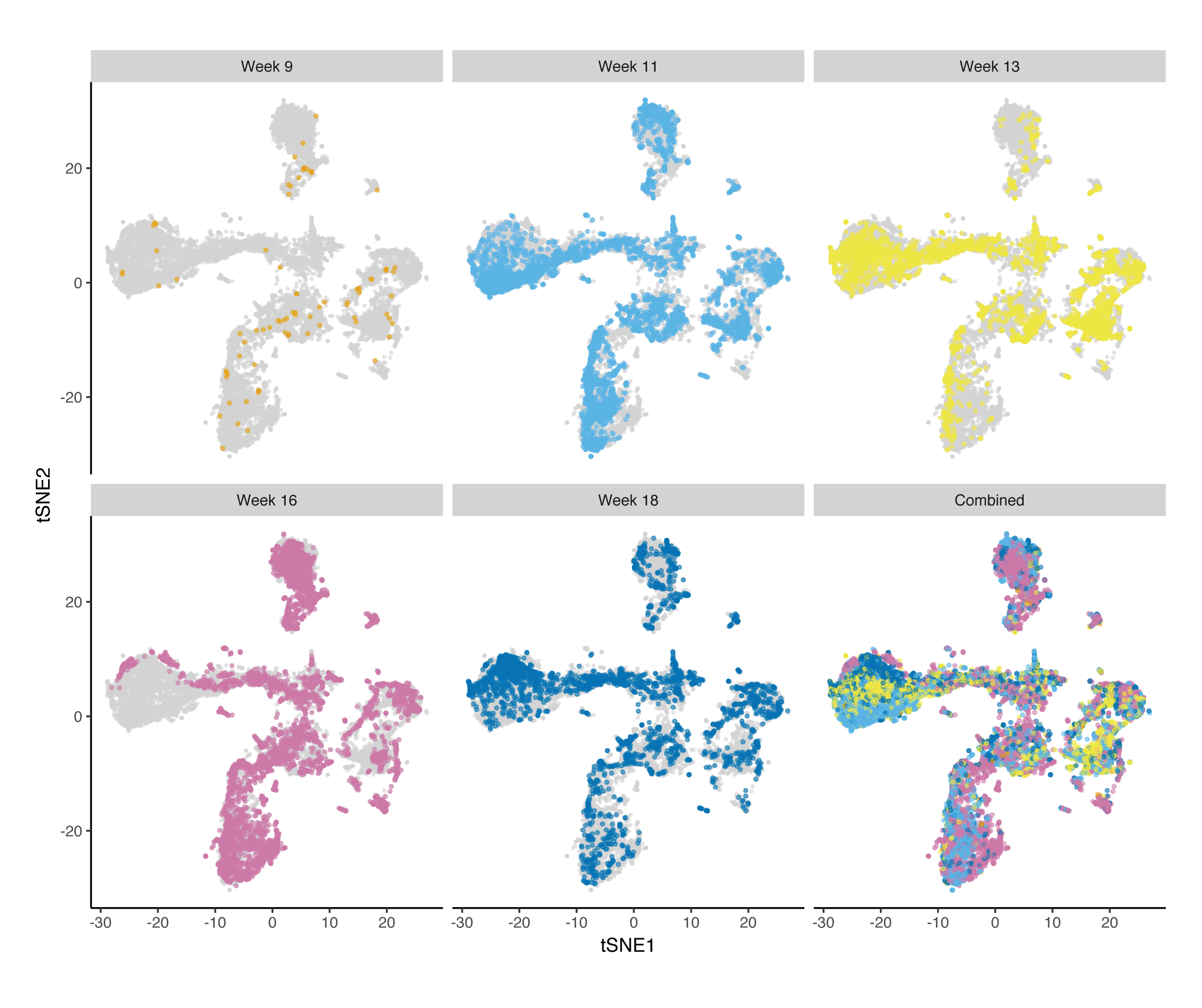

Supplement: S8 Fig — tSNE map calculated for all five samples (w9, w11, w13, w16, w18) combined after batch correction [20]. Developmental age is indicated by color. The numerical data underlying this figure can be found in S1 Data. tSNE, t-distributed stochastic neighbor embedding; w, week. (TIF) [file pbio.3000152.s008.tif]

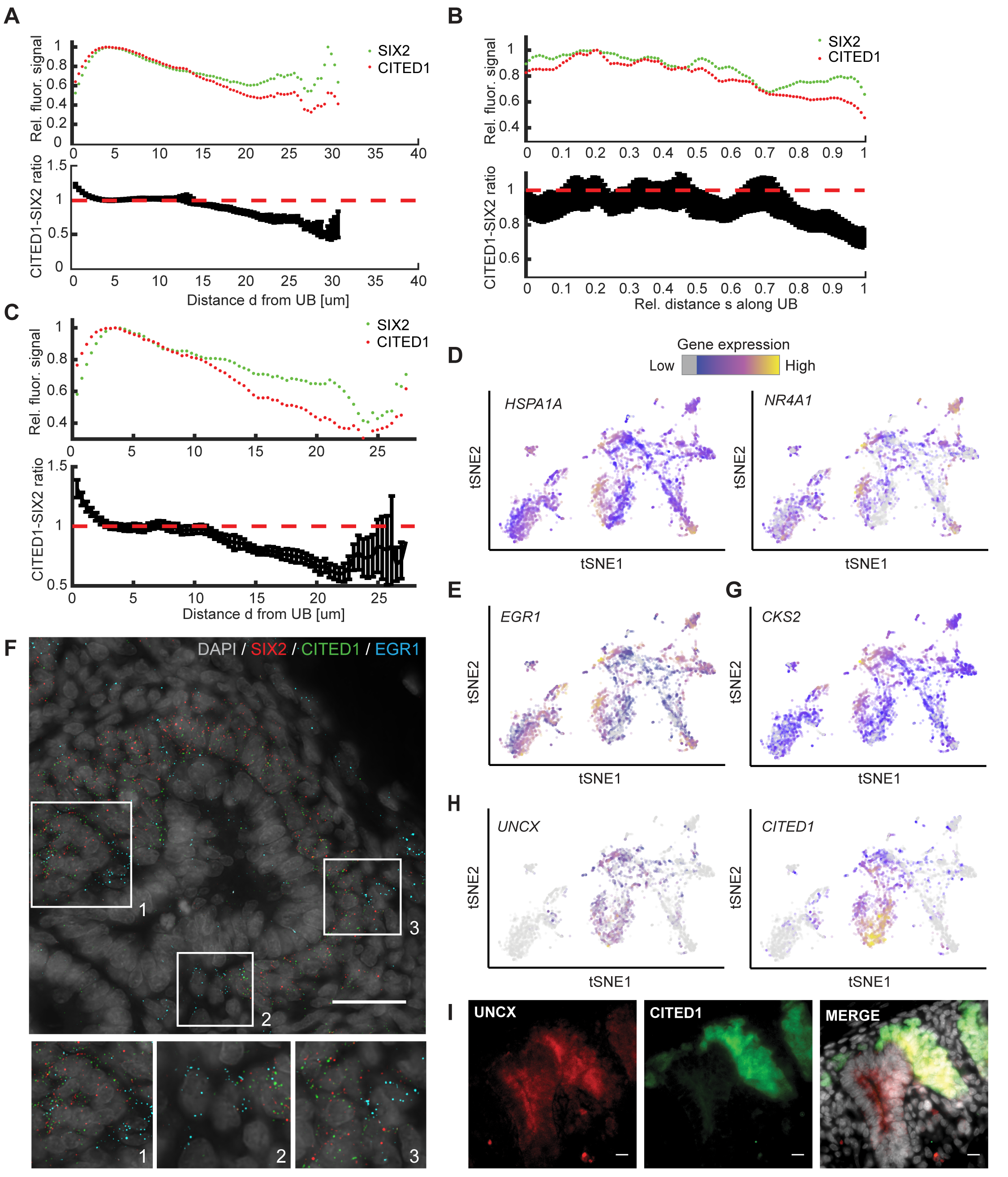

Supplement: S9 Fig — (A and B) Quantification of SIX2 and CITED1 immunostaining with respect to the distance d from UB or distance s along the UB. Compared to the data shown in Fig 5, the fluorophores on the secondary antibodies were swapped. Error bars indicate the SEM calculated over all evaluated profiles (n = 19). (C) Quantification of SIX2 and CITED1 immunostaining with respect to the distance d from UB in which only cells with a relative distance s (along the UB) < 0.2 were taken into account. Error bars indicate the SEM calculated over all evaluated profiles (n = 19). (D) tSNE map showing expression of HSPA1A and NR4A1. Expression is indicated by color; expression values of 1 are plotted in gray. (E) tSNE map showing expression of EGR1. Expression is indicated by color; expression values of 1 are plotted in gray. (F) smFISH of SIX2, CITED1, and EGR1. The three insets at the bottom correspond to the three areas marked by solid boxes in the main image. Scale bar = 25 μm. (G) tSNE map showing expression of CKS2. Expression is indicated by color; expression values of 1 are plotted in gray. (H) tSNE maps showing expression of CITED1 and UNCX. Expression is indicated by color; expression values of 1 are plotted in gray. (I) Immunostaining of CITED1 and UNCX. Scale bar = 10 μm. The numerical data underlying this figure can be found in S1 Data. SEM, standard error of the mean; smFISH, single molecule fluorescence in situ hybridization; tSNE, t-distributed stochastic neighbor embedding; UB, ureteric bud. (TIF) [file pbio.3000152.s009.tif]

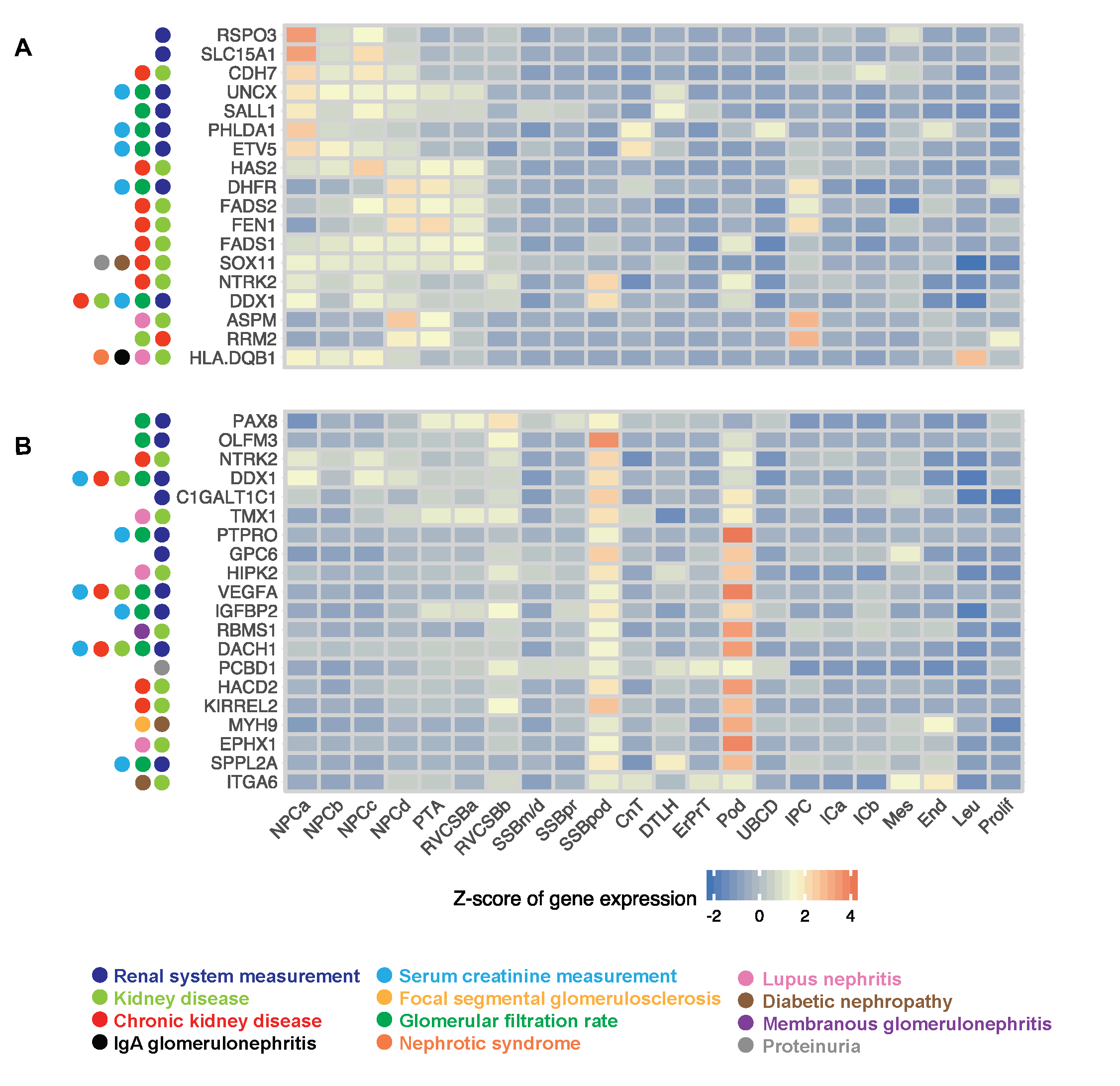

Supplement: S10 Fig — Expression of genes from GWAS traits related to kidney disease. Disease phenotypes associated with these genes are indicated by color; genes were filtered for high expression in cluster(s) of interest relative to all other cell types. Expression was Freeman-Tukey transformed, averaged over all cells in a cluster, and standardized gene-wise. (A) Disease-associated genes expressed in early nephron progenitor states (NPC to PTA). (B) Disease-associated genes expressed in SSBpod. The numerical data underlying this figure can be found in S1 Data. GWAS, genome-wide association studies; NPC, nephron progenitor cell; PTA, pretubular aggregate; SSBpod, s-shaped body podocyte progenitor. (TIF) [file pbio.3000152.s010.tif]
